# Novel genotypes, phenotypes, and triggers in humans with OTULIN haploinsufficiency

**DOI:** 10.70962/jhi.20250018

**Published:** 2025-09-30

**Authors:** Tristan J. van der Linden, Rob J.W. Arts, Catherine M. Biggs, Laleh Habibi, Laura Batlle-Masó, Arjan van Laarhoven, Lisette M. Scheepmaker, Pariya Yousefi, Andrea C. Gómez-Raccio, Zahra Alizadeh, Catharina M. Mulders-Manders, Jaap ten Oever, Janneke Schuurs-Hoeijmakers, Nasrin Alipour-Olyei, Anne Molitor, Daniela Di Giovanni, Raphael Carapito, Seiamak Bahram, Gisela Seminario, Liliana Bezrodnik, Mana Momenilandi, Mohammad Shahrooei, Jacinta Bustamante, Ivona Aksentijevich, Daniel L. Kastner, Mohammad Reza Fazlollahi, Roger Colobran, Stuart E. Turvey, Frank L. van de Veerdonk, Jean-Laurent Casanova, Bertrand Boisson, Bart W. Bardoel, András N. Spaan

**Affiliations:** 1 Department of Medical Microbiology, University Medical Center Utrecht, Utrecht University, Utrecht, Netherlands; 2 Department of Internal Medicine, Radboud Research Institute for Medical Innovation, Radboud Community for Infectious Diseases, Radboud University Medical Center, Nijmegen, Netherlands; 3 Department of Pediatrics, BC Children’s Hospital, The University of British Columbia, Vancouver, Canada; 4 Immunology, Asthma, and Allergy Research Institute, Tehran University of Medical Sciences, Tehran, Iran; 5 Translational Immunology Research Group, Vall d’Hebron Research Institute, Vall d’Hebron Barcelona Hospital Campus, Barcelona, Spain; 6 Servicio de Inmunología, Hospital de Niños Dr. Ricardo Gutiérrez, Buenos Aires, Argentina; 7 Department of Human Genetics, Radboud University Medical Center, Nijmegen, Netherlands; 8 Laboratoire d’ImmunoRhumatologie Moléculaire, INSERM UMR_S 1109, Plateforme GENOMAX, Institut Thématique Interdisciplinaire de Médecine de Précision de Strasbourg, Transplantex NG, Faculté de Médecine, Fédération Hospitalo-Universitaire OMICARE, Fédération de Médecine Translationnelle de Strasbourg, Université de Strasbourg, Strasbourg, France; 9 Service d’Immunologie Biologique, Nouvel Hôpital Civil, Strasbourg, France; 10 Center for Clinical Immunology, Buenos Aires, Argentina; 11 Laboratory of Human Genetics of Infectious Diseases, Necker Branch, INSERM U1163, Necker Hospital for Sick Children, Paris, France; 12 Paris Cité University, Imagine Institute, Paris, France; 13 Department of Microbiology, Clinical and Diagnostic Immunology, Immunology and Transplantation, KU Leuven, Leuven, Belgium; 14 Dr. Shahrooei Lab, Tehran, Iran; 15 Study Center for Primary Immunodeficiencies, Necker Hospital for Sick Children, Paris, France; 16 St. Giles Laboratory of Human Genetics of Infectious Diseases, Rockefeller Branch, The Rockefeller University, New York, NY, USA; 17 Inflammatory Disease Section, National Human Genome Research Institute, Bethesda, MD, USA; 18 Immunology Division, Genetics Department, Vall d’Hebron Research Institute, Vall d’Hebron Barcelona Hospital Campus, Universitat Autònoma de Barcelona, Barcelona, Spain; 19 Department of Pediatrics, Necker Hospital for Sick Children, Paris, France; 20 Howard Hughes Medical Institute, The Rockefeller University, New York, NY, USA

## Abstract

Human OTULIN haploinsufficiency predisposes to life-threatening necrosis of the skin and lungs. Disease is triggered by infectious agents, typically *Staphylococcus aureus*, as well as unknown etiologies. We describe and characterize six unrelated patients who carry rare, predicted deleterious variants of *OTULIN* in heterozygosity. In addition to staphylococcal infections, the disease in the patients is elicited by previously underappreciated triggers, including mechanical or iatrogenic traumas and pseudomonal or clostridial infections. Severe necrosis of the lungs and/or skin are clinical hallmarks of their disease. By combining in vitro allele characterizations and functional studies in patients’ cells, we demonstrate that the patients suffer from OTULIN haploinsufficiency. We provide guidance for assessing heterozygous *OTULIN* variants in diagnostic settings by evaluating in silico measures of predicted deleteriousness. The clinical course of the patients expands the genotypic and phenotypic spectrum of OTULIN haploinsufficiency and provides, in the light of a broadening of triggers, leads for therapeutic interventions.

## Teaser

Human OTULIN haploinsufficiency causes life-threatening staphylococcal disease, but the genotypic and phenotypic spectrum of the disorder remains largely unknown. We describe and characterize six unrelated patients with OTULIN haploinsufficiency, in whom severe necrosis followed infectious and/or traumatic triggers.

## Graphical Abstract

**Figure:**
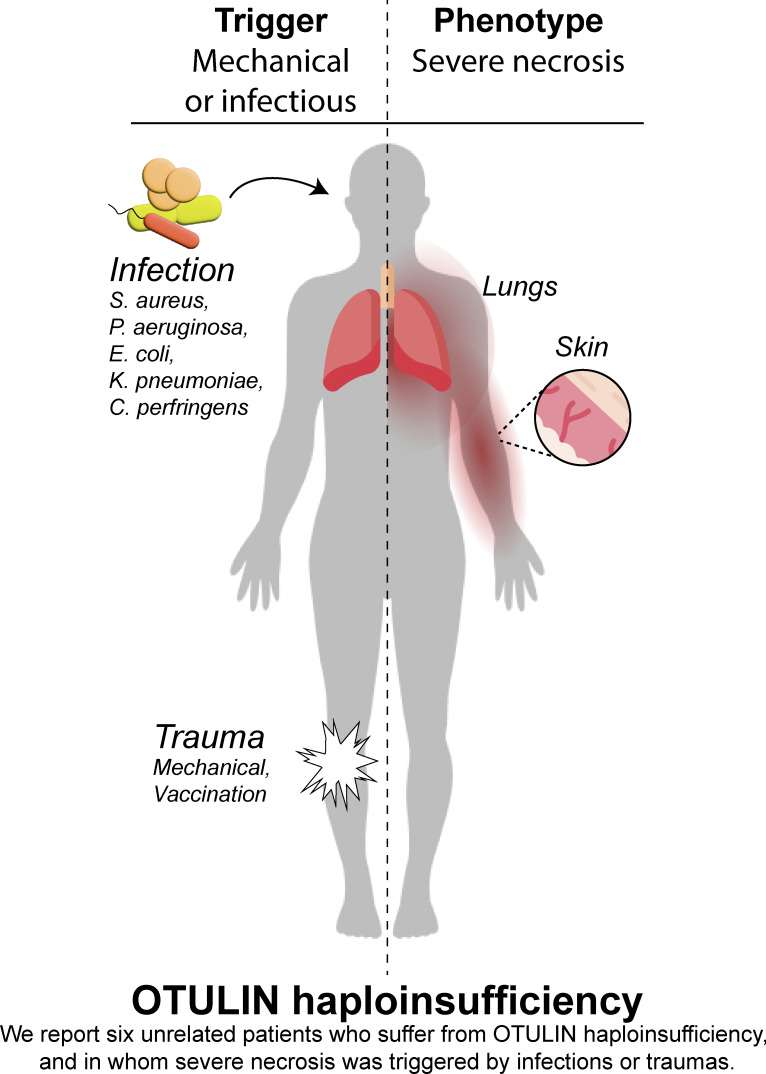

## Introduction

The timely and proportional activation of immune responses is critical for tissue homeostasis and host defense (1). Ubiquitination is a posttranslational modification (PTM) that plays an essential role in dynamically governing diverse cellular processes, including inflammatory and immune signaling pathways (2, 3, 4). Ubiquitination is the process in which polyubiquitin chains are conjugated to substrates. The ubiquitin monomers within these chains are internally linked by their lysine residues or organized in a linear manner using the N-terminal methionine (M1). The mode in which the individual ubiquitin monomers are connected dictates their function. M1-linked polyubiquitination (M1-Ub) is a functionally important PTM governing supramolecular complex formation and inflammatory signaling (5, 6). M1-Ub, also known as linear ubiquitination, is regulated by a dedicated set of enzymes. M1-Ub is assembled by the linear ubiquitin chain assembly complex (LUBAC). This E3 ligase, consisting of the catalytic subunit HOIP and co-activators HOIL1 and SHARPIN, generates M1-Ub chains on substrates such as RIPK1, RIPK2, and NEMO, which is an essential step in the activation of the TNFR1 signaling complex and upregulation of the NF-κB transcription factor complex (7, 8, 9). Conversely, M1-Ub chains are hydrolyzed by the ovarian tumor deubiquitinase domain (OTU) deubiquitinase with linear ubiquitin specificity (OTULIN), a deubiquitinase with exclusive specificity toward M1-Ub (10, 11). By removing M1-Ub, OTULIN attenuates inflammatory signaling in a cell type–specific manner. The C-terminal OTU is the highly conserved catalytic domain, responsible for the hydrolysis of M1-Ub through its conformationally assisted, catalytic triad (11).

Three molecularly distinct OTULIN-related diseases have been reported in humans. Patients carrying biallelic, deleterious mutations of *OTULIN* suffer from a neonatal onset, severe autoinflammatory disorder known as OTULIN-related autoinflammatory syndrome (ORAS) (12, 13, 14, 15, 16). Autoinflammation in ORAS is largely driven by a defective downregulation of NF-κB–dependent signaling in myeloid cells (13, 14). Dominant-negative OTULIN deficiency is an autosomal dominant disorder caused by de novo heterozygous variants encoding antimorphic alleles, in which patients present autoinflammation with subtle features of immunodeficiency (17, 18). Cells from patients suffering from dominant-negative OTULIN deficiency are—like in ORAS—susceptible to TNF-induced cell death (17, 18, 19). Individuals with OTULIN haploinsufficiency (i.e., carriers of a monoallelic loss-of-function allele) are predisposed to necrosis of the skin and/or lungs, triggered by *Staphylococcus aureus* infections and other, as yet unknown, etiologies (20, 21, 22, 23, 24). OTULIN haploinsufficiency is silent for TNF-induced signaling events, and the patients’ blood leukocyte subsets are unaffected. Instead, the disorder underlies susceptibility of nonhematopoietic cells to the *S. aureus* virulence factor α-toxin via the cell type–dependent accumulation of caveolin-1 (20). Caveolin-1 is a scaffolding molecule that regulates the cell surface expression of the α-toxin receptor ADAM10 (25). Caveolin-1 accumulation in the patients’ dermal fibroblasts, but not leukocytes, enhances their susceptibility to α-toxin. OTULIN haploinsufficiency is incompletely penetrant, with variable expressivity and an apparent phenotypic heterogeneity. So far, OTULIN haploinsufficiency has been experimentally proven in 13 patients from nine kindreds (20, 26) and has been suggested in four other patients (21, 22, 23, 24). Penetrance is estimated at around 30% (20). The genotypic and phenotypic spectrum of the disorder and the potential triggers of disease remain, thus, largely unknown.

Here, we describe and characterize six unrelated patients with OTULIN haploinsufficiency, in whom life-threatening necrosis of the skin and/or lungs followed infectious and traumatic triggers. We provide guidance for assessing the clinical significance of heterozygous *OTULIN* variants in diagnostic settings. The clinical course in the patients expands the phenotypic spectrum of OTULIN haploinsufficiency and provides leads for therapeutic interventions.

## Results

### Case reports of the patients studied

We describe six unrelated patients from across the world with severe necrosis of the skin and/or lungs (Table 1). Detailed case reports are provided in the Materials and methods section, and the clinical course in two patients has been reported elsewhere (21, 22). The patients’ first episode of severe disease occurred in childhood or adulthood (their age ranging from 6 to 41 years), with an average age of 27 years at the time of referral. In hindsight, most patients reported probable episodes of disease earlier in their life, albeit of less severe impact. The disease episodes were triggered by infections and/or traumas. In some patients, diagnostic cultures returned pathogenic microorganisms (including *Pseudomonas aeruginosa* in three patients, *Clostridium perfringens* in two patients, members of the Enterobacteriaceae family in two patients, and *S. aureus* in one patient) (Table 1) (22). Apparently noninfectious triggers included skin-compromising traumas (including minor traumas in three patients, vaccination in three patients, and surgery in two patients) (21, 22). Necrosis of the skin and/or lungs was a clinical hallmark of their disease. Skin manifestations included necrotizing cellulitis and abscesses in four patients and a neutrophilic dermatosis consistent with pyoderma gangrenosum in two patients. Four patients suffered from pneumonias, with or without a necrotizing evolution into lung abscesses. The inflammatory profile of the patients required emergency surgical interventions and intensive care unit (ICU) admission in most cases. Routine diagnostic immune profiling (including but not limited to differential leukocyte phenotyping, measurement of immunoglobulin levels, and oxidative burst responses) revealed no overt disturbances (Table S1). At admission, all patients were treated with broad-spectrum antibiotics covering most gram-positive pathogens, with modest or absent clinical response (Table S2). Some patients received immunomodulatory regimens, including steroids and biological therapeutics, with variable results (Table S2). All patients are currently alive.

**Table 1.** Clinical data summary

Patient	Sex	Genotype	Origin	Age referral	Age onset	Reference	Major manifestation	Pathogens identified	Noninfectious triggers
A.II.4	Female	WT/p.A240V	Europe	31 years	4 years	(21)	Necrotizing cellulitis; pathergy-like inflammation; pneumonia; lung abscesses	None identified	Minor trauma
B.III.8	Male	WT/p.E314X	Middle East	6 years	1.5 years	​	Necrotizing cellulitis; skin abscesses; necrotizing panniculitis; pneumonia	*P. aeruginosa*; *Enterobacter* spp.	Vaccination
C.II.3	Male	WT/p.K247RfsX25	South America	16 years	0.5 years	​	Necrotizing cellulitis; skin abscesses; pyoderma gangrenosum; pneumonia	*P. aeruginosa*; *S. aureus*; *C. perfringens*	Vaccination; surgery
D.II.3	Female	WT/p.S351VfsX55	Europe	28 years	28 years	​	Necrotizing cellulitis; skin abscesses	None identified	Surgery
E.II.8	Female	WT/p.W148X	Europe	41 years	15 years	(22)	Cellulitis; skin abscesses; chorioamnionitis; pyelonephritis	*Klebsiella* spp.; *Pseudomonas* spp.; C. *perfringens*; *E. coli*	Minor trauma
F.II.3	Male	WT/c.788G>A (predicted p.R263Q)	North America	38 years	2 mo	​	Soft tissue abscesses; pyoderma gangrenosum; osteomyelitis; inflammatory bowel disease; pneumonia	*S. epidermidis*	Minor trauma; vaccination; surgery

Patient characteristics and disease manifestations of the patients reported. WT, wild-type.

### The patients carry heterozygous variants of OTULIN

**Figure 1.**
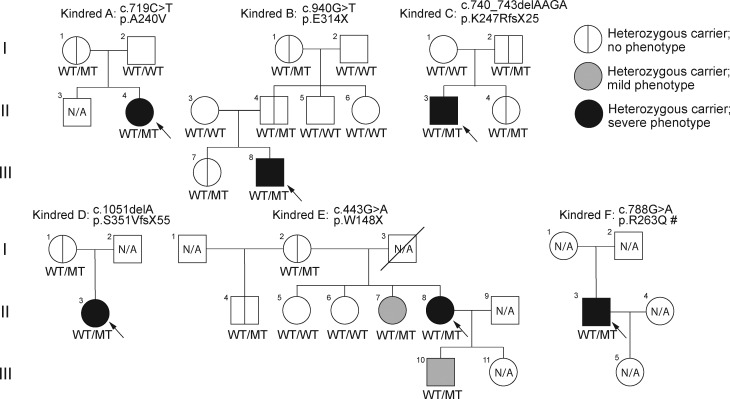
**Pedigrees of patients with heterozygous mutations of *OTULIN*.** Pedigrees of the six unrelated kindreds presenting severe necrosis of the skin and/or lungs following infectious or traumatic triggers and carrying heterozygous mutations of *OTULIN*. WT: wild-type allele; MT: mutant allele. The severity of disease is indicated by the color code assigned to the individual family members. #: The variant carried by patient F.II.3 (c.788G>A) is predicted by Human Genome Variation Society nomenclature as p.R263Q but flagged by SpliceAI for the introduction of a potential splice acceptor site two base pairs from the variant.

Given the severity of their disease and because of a suspicion of an underlying inborn error of immunity (IEI), diagnostic whole-exome sequencing (WES) was performed in the probands (21, 22). Panel analysis of genes, the deficiencies of which are known to underly IEIs (1, 27), revealed rare, predicted deleterious, heterozygous mutations of *OTULIN* in all six patients: two predicted nonsense mutations (c.940G>T/p.E314X and c.443G>A/p.W148X), two predicted frameshift mutations (c.740_743delAAGA/p.K247RfsX25 and c.1051delA/p.S351VfsX55), and two predicted missense mutations (c.719C>T/p.A240V and c.788G>A/p.R263Q) (Fig. 1 and Table 1; case reports in Materials and methods). Two variants (c.443G>A/p.W148X, and c.719C>T/p.A240V) were reported elsewhere (21, 22) but were not characterized. The variant found in patient F.II.3 (c.788G>A) was reported before in another, unrelated patient and characterized as a missense allele (p.R263Q) (20), while all other five alleles are uncharacterized. No potentially pathogenic variants fitting the known modes of inheritance in any of the other genes implicated in IEIs were found. Segregation analyses in the six kindreds were consistent with a dominant inheritance of the *OTULIN* alleles. In the relatives for whom data were available, two heterozygotes expressed similar but milder manifestations (Fig. 1, case reports in Materials and methods). Nine other heterozygous carriers were apparently healthy. Thus, the heterozygous *OTULIN* alleles found in the probands reported here suggest an inborn predisposition to triggered, life-threatening necrosis of the skin and/or lungs, with incomplete penetrance, variable expressivity, and a degree of phenotypic heterogeneity.

### The OTULIN alleles are very rare and deleterious

**Figure 2.**
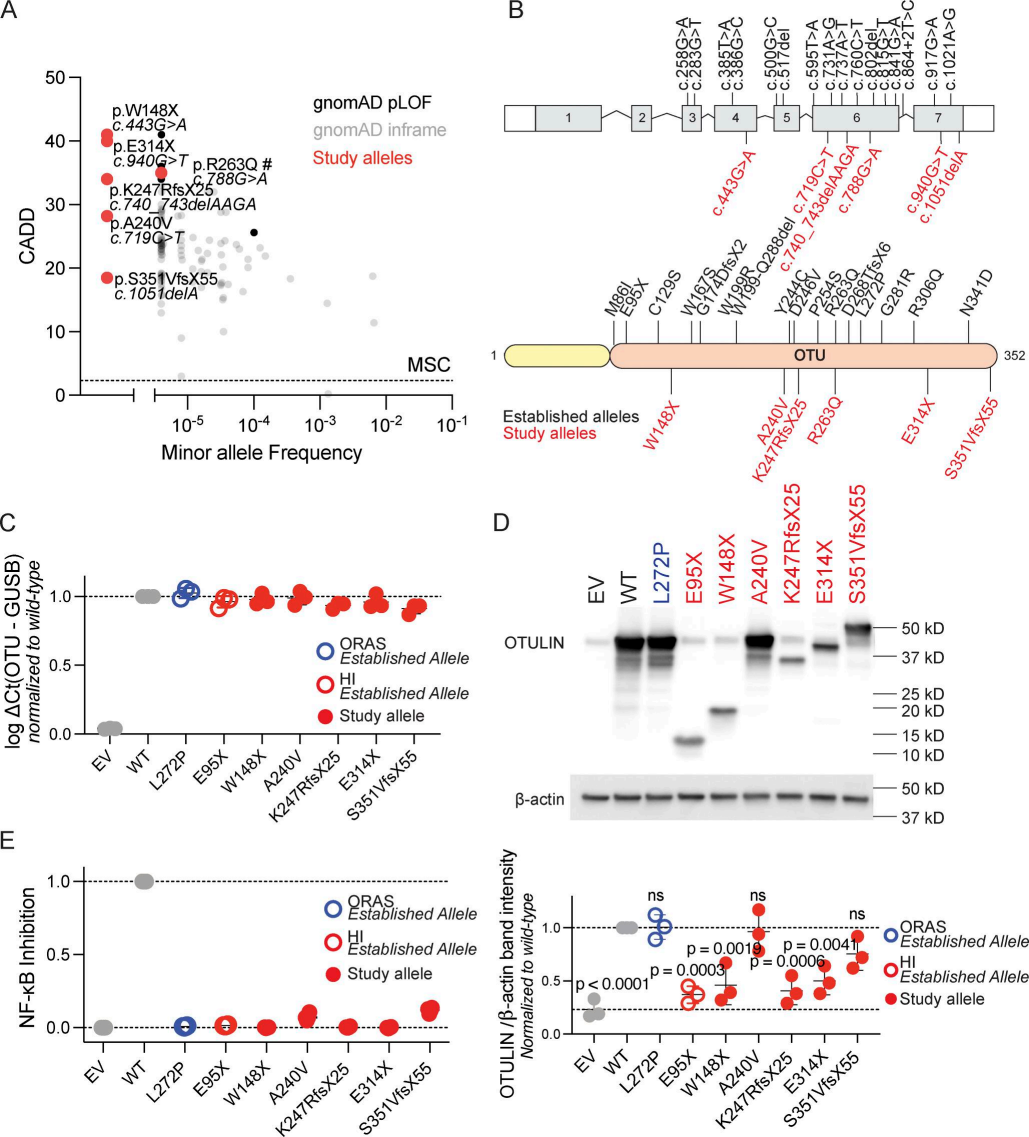
***OTULIN* population genetics and allele characterization. (A)** Population genetics of *OTULIN*, presenting the MAF of *OTULIN* variants reported in the gnomAD database (v4.1.0), against the CADD scores and the mutation significance cutoff (MSC, dotted line). #: The variant carried by patient F.II.3 (c.788G>A) is predicted by Human Genome Variation Society nomenclature as p.R263Q but flagged by SpliceAI for the introduction of a potential splice acceptor site two base pairs from the variant. **(B)** Schematic overview of the OTULIN variants carried by the patients (study alleles, in red) and experimentally proven deleterious OTULIN variants reported elsewhere (established alleles, in black). **(C)***OTULIN* mRNA transcript levels in transiently transfected HEK293T cells, assessed through quantitative reverse transcription PCR (RT-qPCR). *OTULIN* mRNA transcript levels are shown relative to *GUSB* mRNA transcript levels, normalized to transcript levels in cells transfected with wild-type *OTULIN*. Each dot represents the mean of a technical duplicate, *n* = 3 biological replicates. Bars represent mean ± SEM. **(D)** OTULIN expression in transiently transfected HEK293T cells, assessed through western blotting. Shown blot is a representative image, with the plot displaying the relative band intensities of OTULIN over β-actin in *n* = 3 biological replicates, normalized to the relative band intensity in cells transfected with wild-type (WT) OTULIN. Bars represent mean ± SD. Statistical significance was calculated by analysis of variance (ANOVA) with Dunnett’s post hoc correction for multiple comparisons, using samples transfected with WT OTULIN as the comparator. **(E)** NF-κB inhibitory capacity of OTULIN variants, as assessed through an NF-κB–promotor regulated dual-luciferase reporter system in transiently transfected HEK293T cells stimulated with 100 ng/ml TNF. The firefly luciferase over *Renilla* luciferase fold change was inverted, blank corrected to unstimulated cells and cells transiently transfected with empty vector (EV), and subsequently normalized to WT OTULIN. Each dot represents the mean of a technical duplicate, with *n* = 4 biological replicates. Bars represent mean ± SD. HI, cells from an OTULIN haploinsufficient patient with an established allele; ORAS, cells from an ORAS patient with an established allele. Source data are available for this figure: SourceData F2.

**Figure S1.**
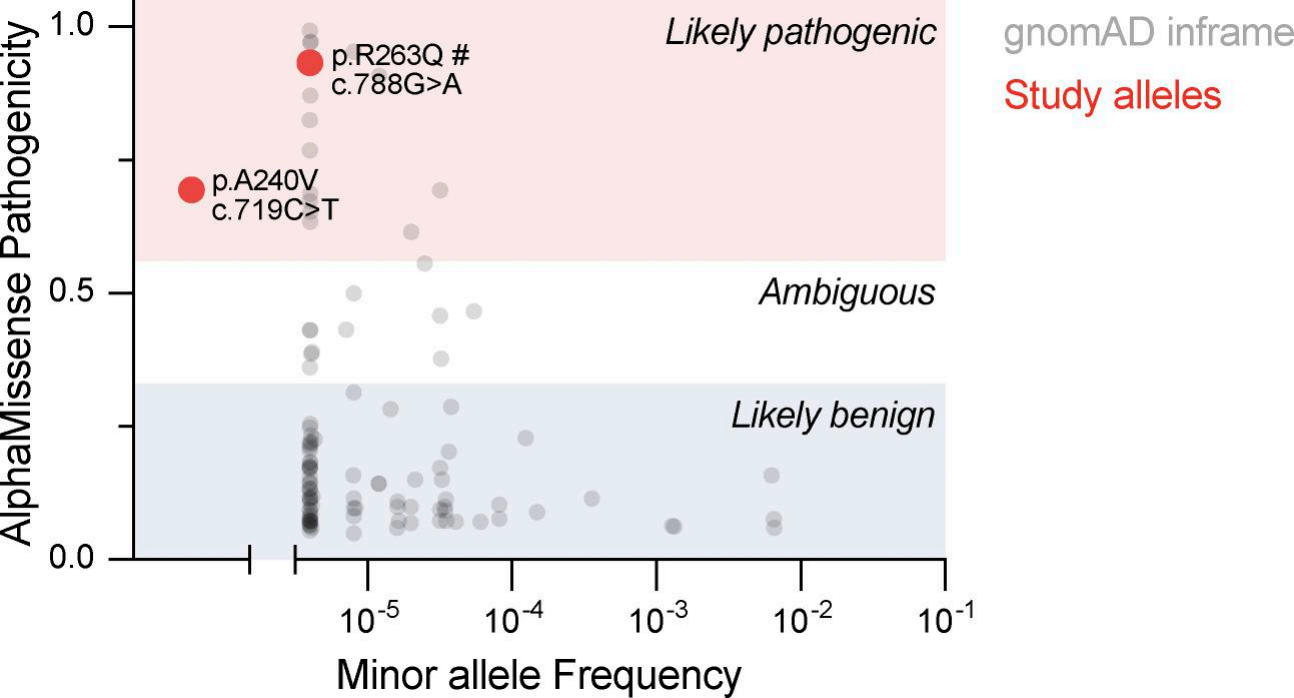
***OTULIN* population genetics.** MAF of missense variants of OTULIN reported in the gnomAD database (v4.1.0) are presented against their AlphaMissense scores. #: The variant carried by patient F.II.3 (c.788G>A) is predicted by Human Genome Variation Society nomenclature as p.R263Q but flagged by SpliceAI for the introduction of a potential splice acceptor site two base pairs from the variant.

**Figure S2.**
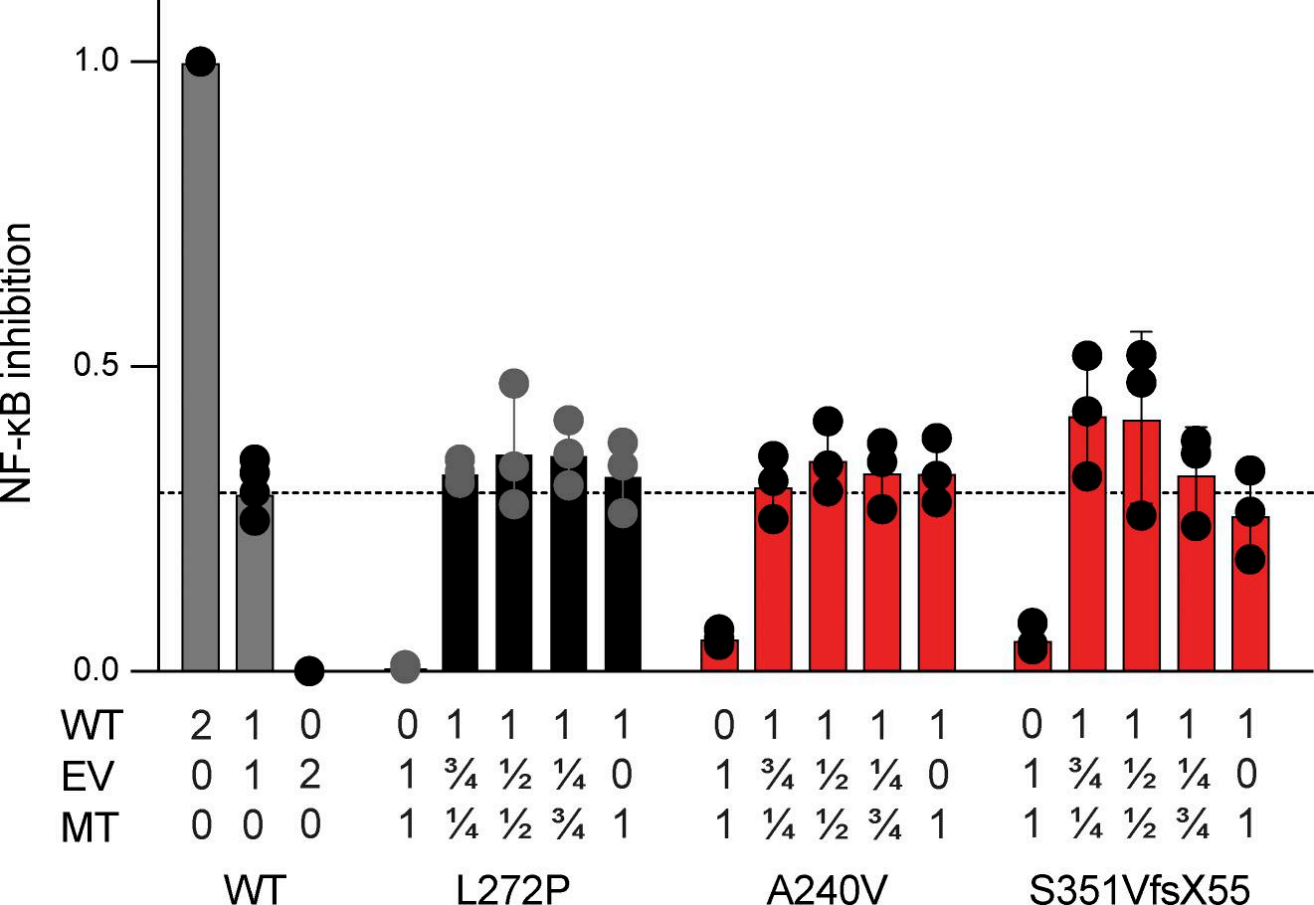
**No negative dominance of expressed patients’ OTULIN alleles.** NF-κB inhibitory capacity of expressed patients’ OTULIN alleles, as assessed through a dual-luciferase assay in HEK293T cells stimulated with 100 ng/ml TNF, which were transiently co-transfected with the indicated OTULIN variants, NF-κB–driven firefly luciferase reporter plasmid, and *Renilla* control plasmid. The firefly luciferase over *Renilla* luciferase fold change was inverted, blank corrected to unstimulated cells and cells transfected only with empty vector (EV), and subsequently normalized to cells transfected only with wild-type (WT) OTULIN. Each dot represents the means of a technical duplicate, with *N* = 3 biological replicates. Bars represent mean ± SD. The numbers in the x-axis represent the ratios of EV, WT, and mutant (MT) OTULIN-encoding vectors. The dotted line indicates activity of cells transfected with one unit of wild-type OTULIN-encoding vector.

*OTULIN* is subject to negative selection pressure (28, 29). Indeed, and consistent with their severe clinical phenotype, the *OTULIN* alleles found in the patients are ultra-rare or not reported at all in the Genome Aggregation Database (gnomAD) (30). The patients’ alleles are predicted to be deleterious, as their combined annotation-dependent depletion (CADD) scores are well above the mutation significance cutoff score (Fig. 2 A) (31, 32). Using the missense variant effect predictor tool AlphaMissense, the allele (c.719C>T/p.A240V) identified in patient A.II.4 is predicted to be pathogenic (Fig. S1) (33). The allele found in patient F.II.3 (c.788G>A), the impact of which was previously interpreted and characterized as a missense variant (p.R263Q) (20), is flagged by contemporary computational tools for the introduction of a potential splice acceptor site two base pairs from the variant (SpliceAI delta score: 0.91) (34). All variants are located in the catalytic OTU of the protein (Fig. 2 B). To characterize the functional impact of the p.A240V, p.E314X, p.K247RfsX25, p.S351VfsX55, and p.W148X alleles, we overexpressed the corresponding cDNAs in HEK293T cells. In overexpression, mRNA transcript levels were like those of wild type for all variants. The p.A240V variant was expressed at normal protein levels when compared with the wild-type allele, like the known pathogenic ORAS-associated p.L272P variant (14). Protein expression of the p.S351VfsX55 variant, which is located in the last exon and may thus impact nonsense mediated mRNA decay, was slightly reduced (Fig. 2, C and D). In contrast, the nonsense (p.W148X, p.E314X) and frameshift (p.K247RfsX25) variants were loss-of-expression at protein levels (Fig. 2, C and D). We subsequently assessed the capacity of the gene products to inhibit NF-κB signaling following stimulation with TNF. This functional assay was previously shown to effectively distinguish pathogenic and nonpathogenic variants (20). All patients’ *OTULIN* alleles were severely hypomorphic or amorphic (Fig. 2 E). We found no negative dominance for the expressed alleles (Fig. S2). Thus, the patients reported here suffer from autosomal dominant OTULIN deficiency by means of haploinsufficiency.

### OTULIN expression is strongly reduced in the patients’ cells

**Figure 3.**
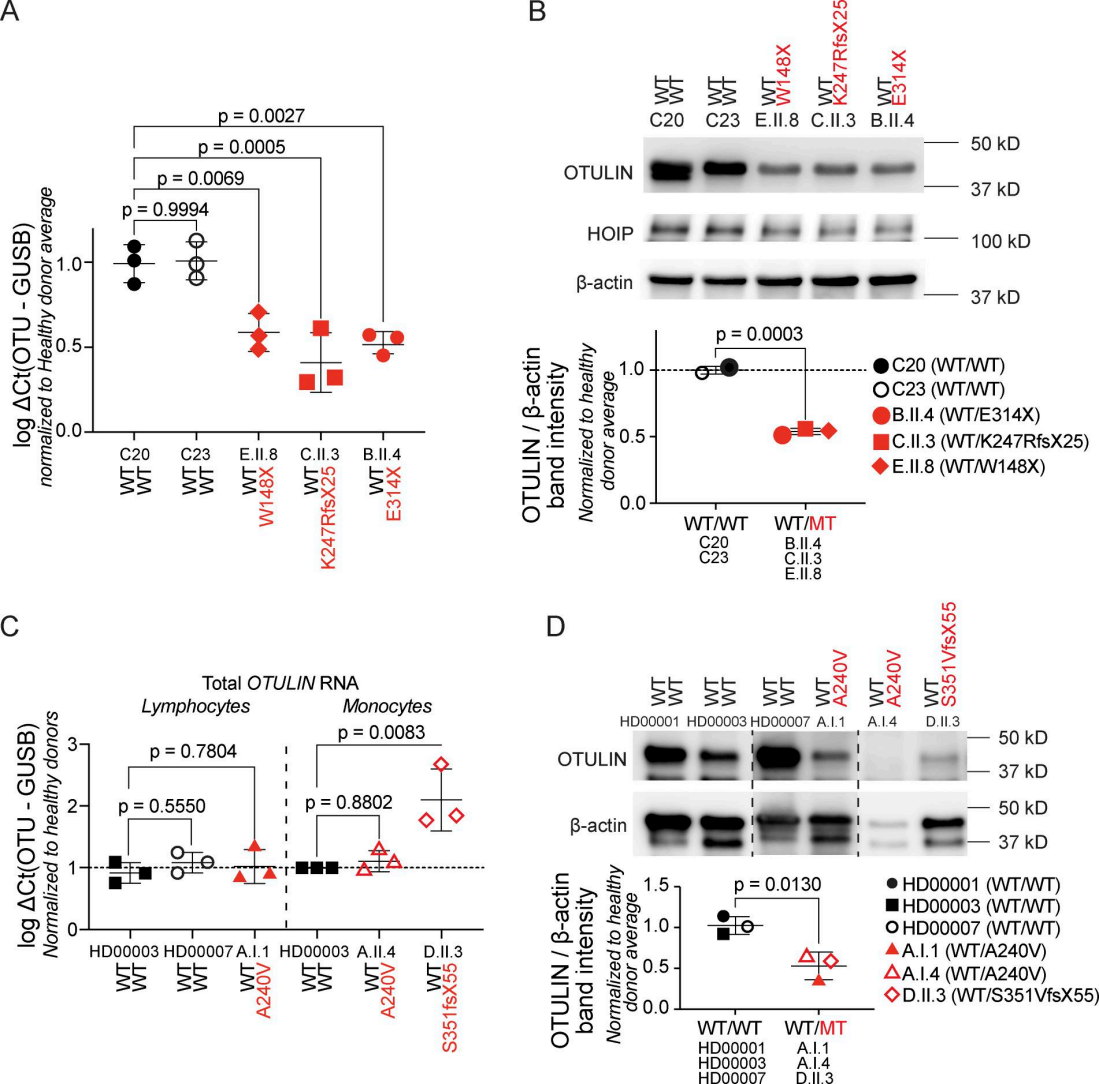
**OTULIN expression in the patients’ cells. (A)** *OTULIN* mRNA transcript levels in PDFs, assessed through RT-qPCR. *OTULIN* mRNA transcript levels are shown relative to *GUSB* mRNA transcript levels, normalized to the average of both healthy donor transcript levels. Each dot represents the means of a technical duplicate, with *N* = 3 replicates per patient. Bars represent mean ± SEM. Statistical significance was calculated by analysis of variance (ANOVA) with Dunnett’s post hoc correction for multiple comparisons, using samples from healthy donors as the comparator. **(B)** Expression of OTULIN and HOIP as determined by western blotting in the patients’ PDFs. Shown blot is a representative image, with the plot displaying the relative band intensity of OTULIN over β-actin, where each dot represents the mean of *N* = 3 replicates per patient, normalized to the average of both healthy donor expression levels. Bars represent mean ± SD. Statistical significance was calculated by unpaired *T* tests. **(C)***OTULIN* mRNA transcript levels in the patients’ lymphocytes and monocytes, assessed through RT-qPCR. *OTULIN* mRNA transcript levels are shown relative to *GUSB* mRNA transcript levels, normalized to the average of healthy donor transcript levels. Each dot represents the mean a technical duplicate normalized to the average of healthy donor transcript levels, with *N* = 3 replicates per patient. Bars represent mean ± SEM. Statistical significance was calculated by ANOVA with Dunnett’s post hoc correction for multiple comparisons, using samples from healthy donors as the comparator. **(D)** OTULIN expression in the patients’ PBMCs, assessed through western blotting. Shown blot is a representative image, with the plot displaying the relative band intensity of OTULIN over β-actin. Each dot represents the mean of *N* = 2 replicates per patient. Bars represent mean ± SD, and statistical significance was calculated by unpaired *T* tests. WT/WT, healthy donors; WT/MT, OTULIN haploinsufficient patients; MT, mutant; WT, wild-type; RT-qPCR, quantitative reverse transcription PCR. Source data are available for this figure: SourceData F3.

**Figure 4.**
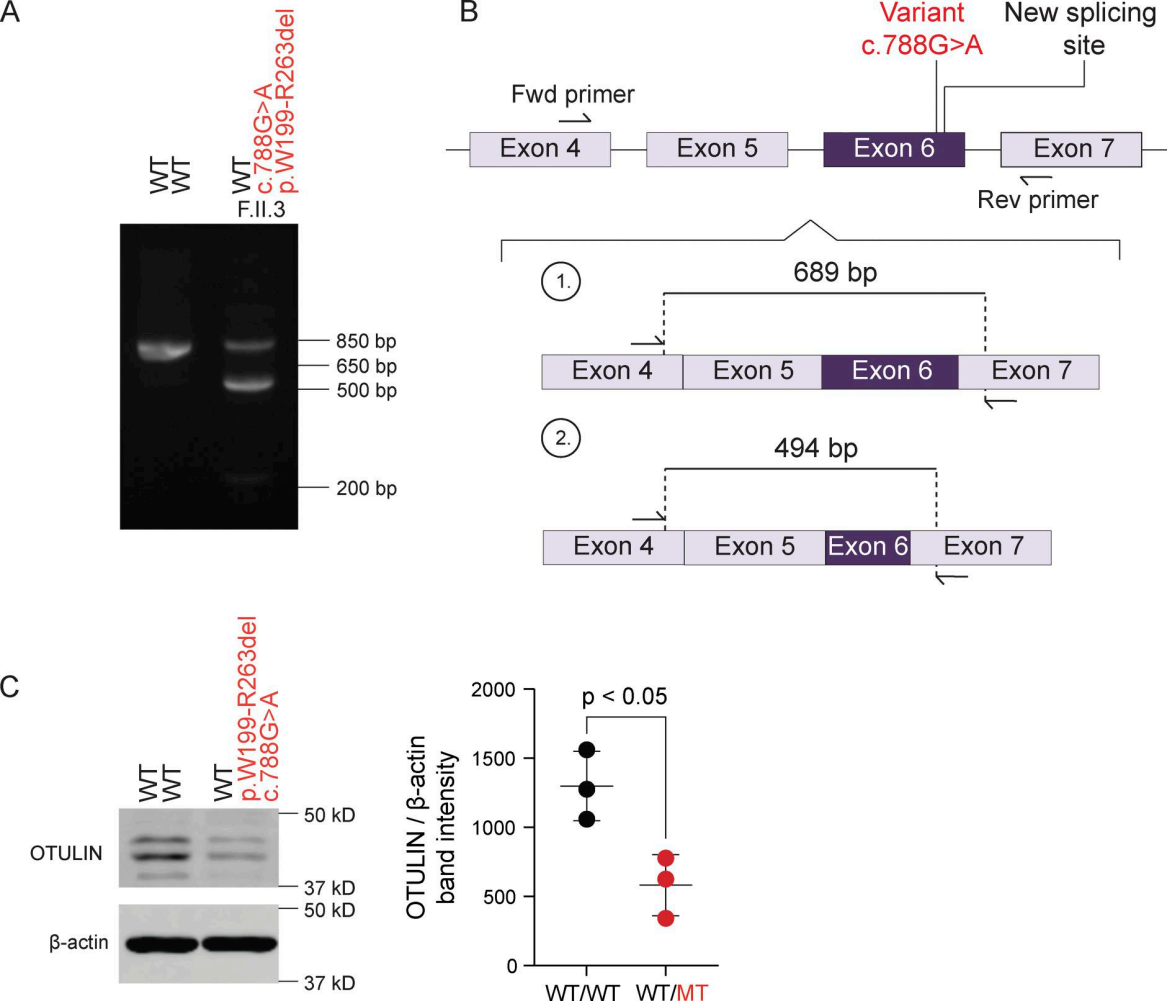
**Aberrant *OTULIN* splicing in patient F.II.3. (A)** Reverse transcription PCR amplification of *OTULIN* mRNA spanning the variant site carried in heterozygosity in patient F.II.3. **(B)** Schematic representation of the altered mRNA transcript induced by the novel splice acceptor in patient F.II.3. **(C)** OTULIN expression relative to β-actin in whole-cell lysates of T cells from patient F.II.3 in comparison with those from a healthy control. Shown blot is a representative image, with the plot displaying the relative band intensity of OTULIN over β-actin with *N* = 3 biological replicates. Bars represent mean ± SD. Statistical significance was calculated by an unpaired *T* test. WT/WT, healthy donors; WT/MT, OTULIN haploinsufficient patient; MT, mutant; WT, wild-type. Source data are available for this figure: SourceData F4.

Cellular overexpression assays characterize the functional capacities of OTULIN variants but tend to overestimate the levels of endogenous expression (14, 20). In primary dermal fibroblasts (PDFs) from patients carrying nonsense or frameshift alleles (patients B.II.4, C.II.3, and E.II.8), the total levels of *OTULIN* mRNA transcripts and the total OTULIN protein levels were approximately half when compared with those in cells from healthy controls (Fig. 3 A). These observations point toward instability of the transcripts at the mRNA levels. Consistent with prior findings, expression of the LUBAC component HOIP was normal in the patients’ PDFs (Fig. 3 B). In lymphocytes and monocytes from patients A.I.1 and D.II.3, of whom no PDFs were available, total *OTULIN* mRNA transcript levels appeared not to be reduced (Fig. 3 C). Total OTULIN protein levels were reduced when comparing the patients’ peripheral blood mononuclear cells (PBMCs) with cells from healthy donors (Fig. 3 D), suggesting protein instability. Targeted polymerase chain reactions (PCRs) on cDNA generated from patient’s F.II.3 peripheral blood revealed an abnormal amplicon size, indicating aberrant splicing (Fig. 4 A). Sanger sequencing demonstrated that the full-length band contained wild-type cDNA, and a second smaller band contained cDNA with a partial deletion of exon 6 (Fig. 4 B). The resulting deletion of 195 nucleotides at the transcript level corresponds with a deletion of 65 amino acids at the protein level (p.W199-R263del). This patient’s allele was previously characterized as a severely hypomorphic missense variant (p.R263Q) in an unrelated patient (20). In both patients, total OTULIN protein levels were approximately half when compared with those in cells from healthy controls (20), and no OTULIN protein variants with a differential molecular weight size were observed (Fig. 4 C). Thus, the overall functional capacity of OTULIN in the patients is approximately half when compared with healthy controls and consistent with OTULIN haploinsufficiency.

### Functional consequences of OTULIN haploinsufficiency

**Figure 5.**
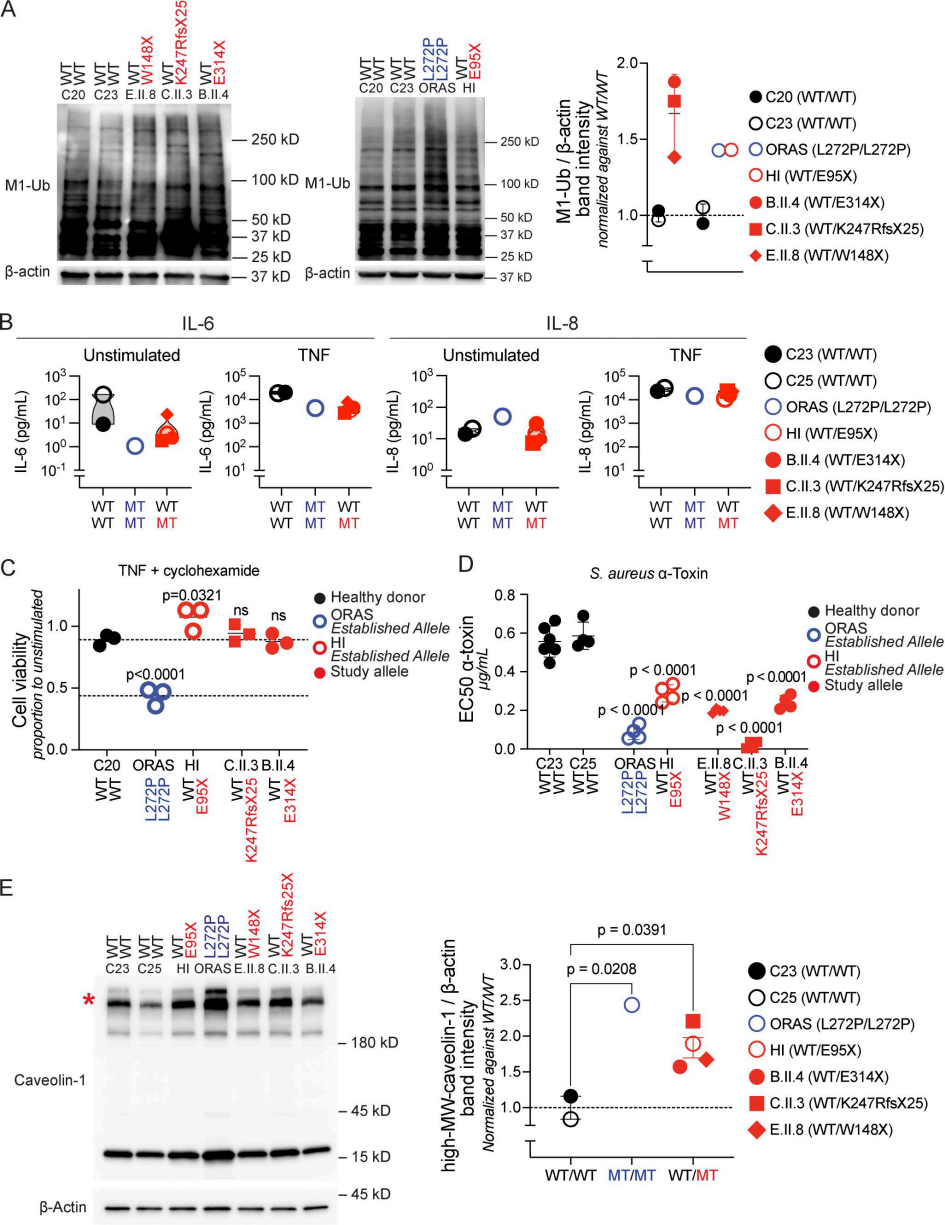
**Functional consequences of OTULIN haploinsufficiency. (A)** Accumulation of linear ubiquitin (M1-Ub) chains in whole-cell lysates of the patients’ PDFs. Shown blots are representative images, with the plot displaying the relative band intensity of M1-Ub over β-actin. Each dot represents the mean of *N* = 3 replicates per patient, normalized to the relative mean band intensity of M1-Ub over β-actin of both healthy donors. **(B)** Cytokine secretion in the patients’ PDFs, as detected by ELISA. Data points indicate the mean of *N* = 2 independent experiments, with three replicates per patient per experiment. **(C)** Viability of PDFs after 24 h of incubation with TNF (20 ng/ml) and cycloheximide (12.5 ng/ml), relative to unstimulated cells. Statistical significance was calculated through ANOVA with Dunnett’s multiple comparisons test, using samples from a healthy donor as the comparator. **(D)** The half-maximum effective concentration (EC_50_) of α-toxin on PDFs after 24 h of intoxication, as determined through three-parameter nonlinear regression analyses. Statistical significance was calculated through ANOVA with Dunnett’s multiple comparisons test, using samples from healthy donors as the comparator. **(E)** Accumulation of high-molecular weight caveolin-1 in whole-cell lysates of the patients’ PDFs. Shown blot is a representative image, with the plot displaying the relative band intensity of high-MW caveolin-1 over β-actin. Each dot represents the mean of *N* = 3 replicates per patient, normalized to the mean high-MW caveolin-1 over β-actin fold change of both healthy donors. Bars represent mean ± SEM. Statistical significance of differences were calculated through ANOVA with Dunnett’s multiple comparisons test, using samples from healthy donors as the comparator. Asterisk indicates high-molecular weight caveolin-1. HI, established allele in an OTULIN haploinsufficient patient; ORAS, established allele in an ORAS patient. WT/WT, healthy donors; MT/MT, ORAS patient; WT/MT, OTULIN haploinsufficient patients; MT, mutant; WT, wild-type; MW, molecular weight; ANOVA, analysis of variance. Source data are available for this figure: SourceData F5.

**Figure S3.**
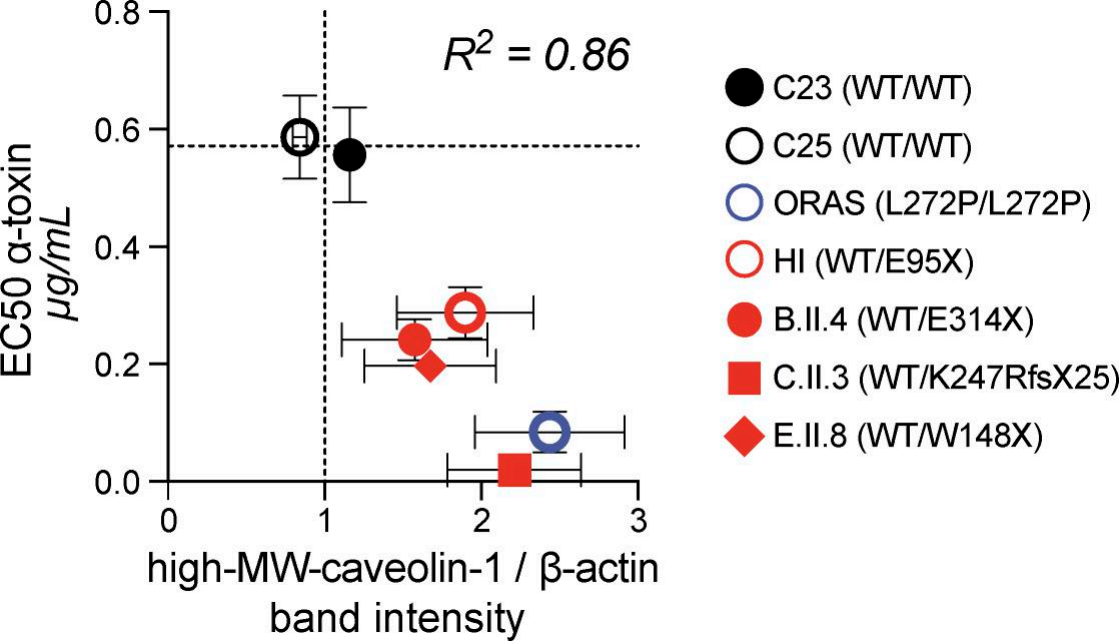
**Functional consequences of OTULIN haploinsufficiency.** Sensitivity of the patients’ PDFs to the staphylococcal α-toxin, expressed as the half-maximum effective concentration (EC_50_) of α-toxin after 24 h of intoxication, plotted against the relative abundance of high-molecular-weight (MW) caveolin-1. Each dot represents the mean of *N* = 3 replicates per patient. The high-MW caveolin-1/β-actin fold change was normalized to the mean of both healthy donors. WT/WT, healthy donors. HI, cells from an OTULIN haploinsufficient patient with an established allele; ORAS, cells from an ORAS patient with an established allele. WT, wild-type.

We next tested the accumulation of M1-Ub in the patients’ PDFs. As expected, overall levels of proteins decorated with M1-Ub were increased in the patients’ cells when compared with controls (Fig. 5 A). By regulating M1-Ub, OTULIN governs cell-intrinsic immunity and inflammatory signal transduction in a genotype and cell type–dependent manner. Instability and degradation of LUBAC in nonhematopoietic cells is a feature of ORAS, but not OTULIN haploinsufficiency (Fig. 3 B) (12, 20), and predisposes to TNF-induced cell death in ORAS patients’ PDFs (12, 13, 14, 19). Indeed, PDFs from patients B.II.4, C.II.3, and E.II.8 displayed no defects in IL-6 and IL-8 secretion (Fig. 5 B) and were, in contrast to cells from an ORAS patient, not susceptible to stress-induced cell death upon exposure to TNF (Fig. 5 C). Instead, OTULIN haploinsufficiency underlies susceptibility of nonhematopoietic cells to the staphylococcal virulence factor α-toxin (20). Consistent with prior observations, PDFs from patients B.II.4, C.II.3, and E.II.8 showed an increased susceptibility toward cell death induced by α-toxin when compared with PDFs from healthy controls (Fig. 5 D). This cell-intrinsic defect of immunity against α-toxin results from the OTULIN-dependent accumulation of caveolin-1, a scaffolding molecule in the cell membrane (20, 25). Indeed, SDS-resistant high-molecular weight caveolin-1–containing complexes accumulated in whole-cell lysates from the patients’ PDFs (Fig. 5 E). The accumulation of caveolin-1 complexes correlated with the susceptibility of the patients’ PDFs to α-toxin induced cell death (Fig. S3). These data, thus, confirm the molecular mechanism underlying staphylococcal disease in OTULIN haploinsufficiency. Other microbial and traumatic triggers might act by a similar mechanism, as suggested by the newly reported patients who were not diagnosed with *S. aureus* infections (21, 22).

### Prediction of alleles

**Figure 6.**
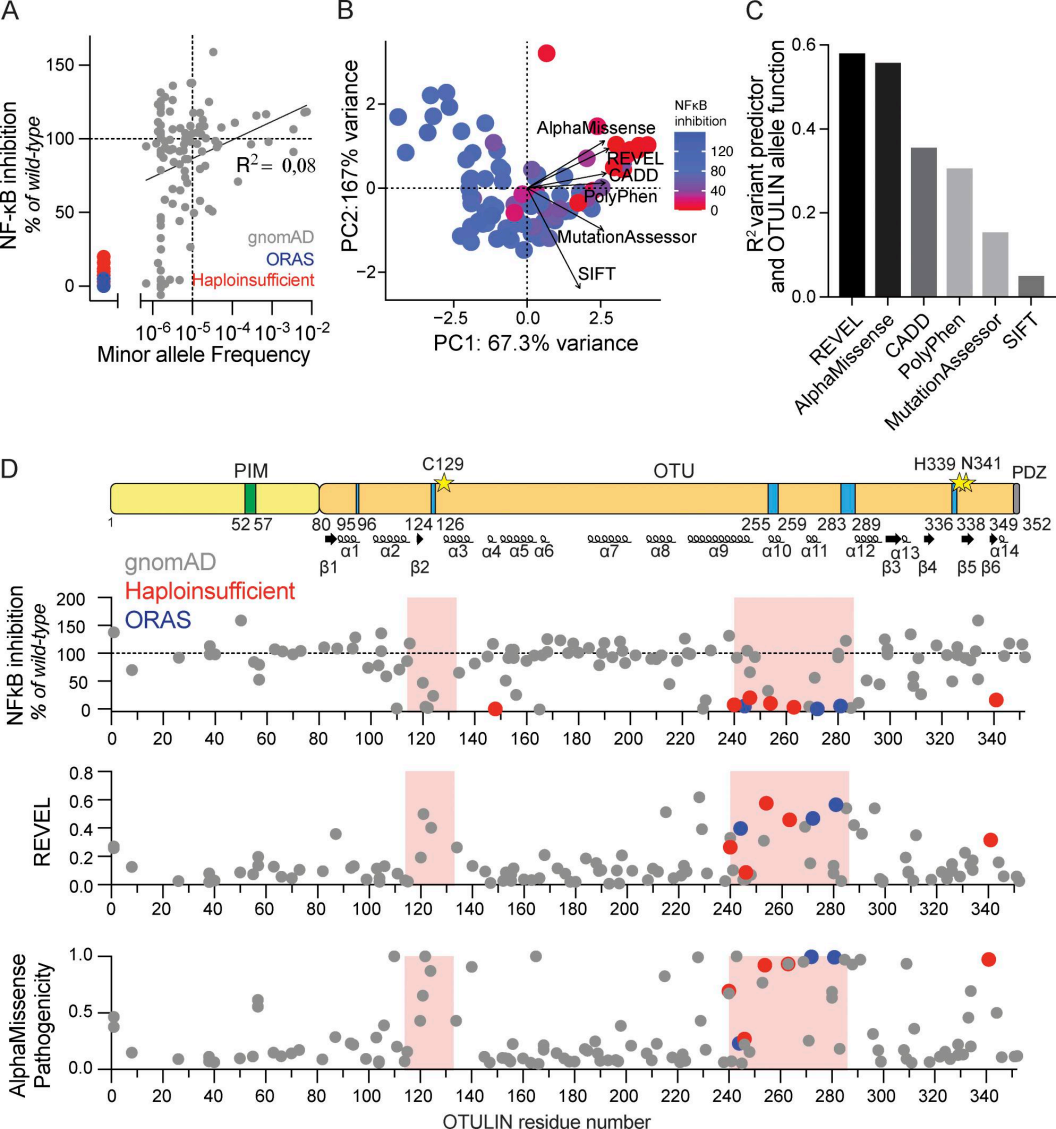
**Predicted deleteriousness of *OTULIN* alleles. (A)** The NF-κB inhibitory capacity of *OTULIN* variants reported in gnomAD v.2.1.1 plotted against their respective MAFs (20). The solid line shows the linear correlation, as calculated through simple linear regression. The horizontal dashed line indicates the activity of the wild-type allele. The vertical dashed line indicates the best-performing MAF cutoff, as determined through a ROC analysis. **(B)** Analyses of various in silico tools for the prediction of the deleteriousness of *OTULIN* missense variants with MAFs <1 × 10^−5^ by means of a principal component analysis. **(C)** Regression analyses of the experimentally derived NF-κB inhibitory capacities of missense variants in *OTULIN* reported in gnomAD v.2.1.1 against a panel of variant pathogenicity predictors. Analyses are based on allele activities described elsewhere (20). **(D)** N- to C-terminal plotted representation of experimentally derived NF-κB inhibitory capacities and REVEL scores of all missense variants of OTULIN reported in gnomAD v2.1.1. The schematic representation of the protein is to the same scale as the x-axes of the plots, with the PUB-interacting motif (PIM) in green, the OTU in salomie, the PDZ-binding motif (PDZ) in grey, the M1-Ub–binding sites in cyan, the catalytic residues highlighted with stars, and the secondary structures annotated. In the plots, the red bars indicate the regions in OTULIN enriched in deleterious mutations. Grey dots indicate missense variants reported in gnomAD, blue dots indicate experimentally validated alleles reported in homozygosity in ORAS patients, and red dots indicate experimentally validated alleles reported in heterozygosity in OTULIN haploinsufficient patients.

**Figure S4.**
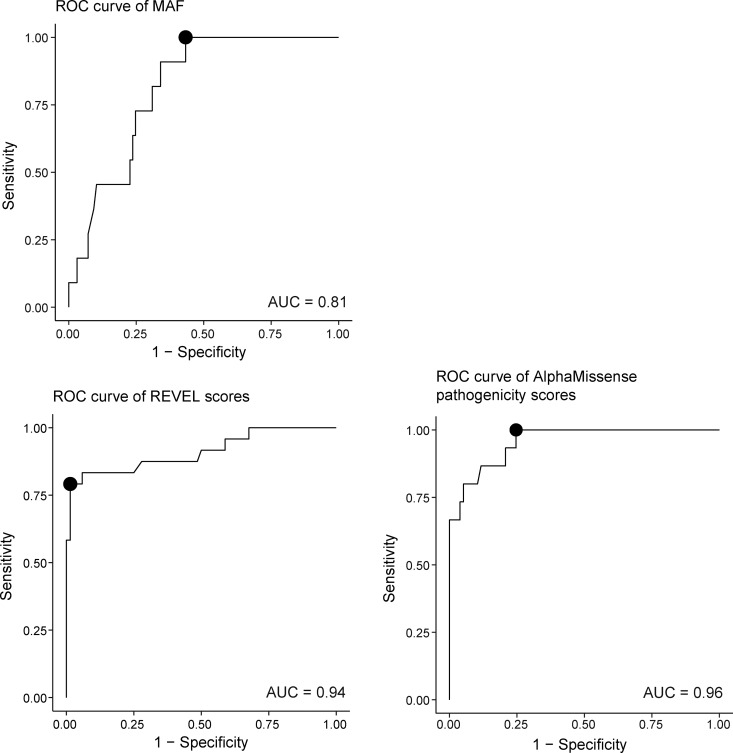
**ROC curve for the prediction of OTULIN variant deleteriousness.** ROC curves for the prediction of OTULIN variant deleteriousness through the MAF, REVEL, or AlphaMissense scores. The dot represents the cutoff values (1 × 10^−5^ for MAF; 0.27 for REVEL; 0.23 for AlphaMissense) recommended for the prediction of OTULIN variant deleteriousness. These values were determined by maximizing the sum of sensitivity and specificity, with a classification system where allele activities equal to or lower than 25% of wild-type were labeled as deleterious.

Because of incomplete penetrance, variable expressivity, and phenotypic heterogeneity in OTULIN haploinsufficiency, interpretation of next-generation sequencing data currently requires experimental validation of uncharacterized alleles. Predicted loss-of-function (pLOF) variants with minor allele frequencies (MAFs) <1 × 10^−5^ are all amorphic (20), and pLOF variants not reported in gnomAD are, therefore, likely to be deleterious when observed in patients. Because the interpretation of missense alleles without experimental validation is more challenging, we tested the use of in silico tools for predicting deleteriousness of missense variants experimentally assessed elsewhere (20). We compared the allele activities of missense variants reported in gnomAD (20, 30) with their MAFs and a panel of variant pathogenicity predictors (CADD, AlphaMissense, SIFT, PolyPhen-2, REVEL, and MutationAssessor) (Table S3) (31, 33, 35, 36, 37, 38). Overall, the MAF showed a poor correlation with experimentally proven deleteriousness, with a best-performing cutoff at 1 × 10^−5^ (Fig. 6 A and Fig. S4) (20). Indeed, all amorphic and hypomorphic variants (with <25% wild-type levels of activity) had MAFs <1 × 10^−5^ (Fig. 6 A). Thus, missense variants with MAFs ≥1 × 10^−5^ are unlikely to cause OTULIN haploinsufficiency. To determine the best predictor for missense variants with MAFs <1 × 10^−5^, we performed principal component and regression analyses (Fig. 6, B and C). REVEL and AlphaMissense were the best performing models (Fig. 6, B and C; and Fig. S4). By plotting the functional impact of missense alleles across the protein, we found a clustering of deleterious variants in two regions (Fig. 6 D): one (residues 114–133) in proximity of the catalytic site C129 and another (residues 240–286) in a region containing multiple M1-Ub–binding sites (11). Thus, very rare missense variants found in these domains are particularly prone for deleteriousness, as demonstrated by the p.A240V allele found in patient A.II.4. Collectively, the assessment of MAFs, REVEL or AlphaMissense scores, and domains affected can assist in the interpretation of *OTULIN* variants in the absence of experimental validation.

## Discussion

We describe six unrelated patients with severe necrosis of their skin and/or lungs, who all carry monoallelic, deleterious mutations in *OTULIN*. The genetic mechanism underlying their dominant form of OTULIN deficiency is via haploinsufficiency. We characterize five alleles functionally and describe one allele previously reported in an unrelated patient (20). In line with the patients’ phenotype, these alleles are severely hypomorphic or amorphic. The consequence of one variant, introducing a splicing acceptor site previously interpreted as a missense allele (20), illustrates the limitations of Human Genome Variation Society nomenclature (39) and the potential of artificial intelligence–assisted prediction algorithms (34). In addition to their hypomorphic or amorphic nature, the alleles result in a reduced OTULIN expression in the patients’ cells. Common to all alleles underlying the various OTULIN deficiencies, and consistent with the negative selection pressure exerted on *OTULIN*, disease-causing variants are ultra-rare in the general population. The activity of *OTULIN* alleles can be evaluated in vitro by measuring their capacity to antagonize NF-κB–dependent signaling, but this approach overestimates endogenous protein stability (20). Similarly, cellular assays measuring deubiquitination overestimate the activity of severe hypomorphs (14, 20). Based on the functional assessment of many variants, we propose a stepwise approach guided by the MAF and in silico tools when considering *OTULIN* variants in a diagnostic setting. REVEL and AlphaMissense perform best in predicting severely hypomorphic or amorphic missense variants, but low pathogenicity scores for disease-causing variants illustrate its imperfection (14, 20, 33). Because no in silico algorithms can discriminate hypomorphs from antimorphs, functional assessment and detection of endogenous expression of mutant alleles remain essential in case dominant-negative OTULIN deficiency is suspected.

The relatively high average age at the onset of life-threatening disease in the OTULIN haploinsufficient patients described here—most being well in their second decade of life—is consistent with that in the initial case series reported (20). This late onset of disease contrasts with the very early onset of severe disease in autosomal recessive OTULIN deficiency (12, 13, 14) and dominant-negative OTULIN deficiency (17, 18). Thus, human OTULIN haploinsufficiency is an IEI that typically, but not exclusively, manifests at adult age. Interestingly, and in retrospect, most patients described here reported milder episodes of disease earlier in their lives, with an average age at the onset of subtle manifestations of 8 years. These observations suggest that clinical features of OTULIN haploinsufficiency can remain underrecognized for considerable time. Despite the degree of phenotypic heterogeneity seen in patients with OTULIN haploinsufficiency, the clinical course in the currently known patients demonstrates that the skin and lungs are particularly prone for the development of abscesses and necrosis. Irrespective of the primary organs affected, the episodes of severe necrosis were accompanied by potentially life-threatening inflammation in all patients. All six probands reported here are currently alive, in contrast to the demise of two patients reported earlier (20). In the kindreds described here, and consistent with the kindreds originally reported, clinical penetrance was incomplete, and the expressivity of disease was variable. Future follow-up will have to reveal if expressivity is indeed variable or if relatives with mild phenotypes are prone to develop severe manifestations at a later point in their lives.

In all currently known patients suffering from OTULIN haploinsufficiency, including the patients reported here, severe necrotic disease followed infectious and/or traumatic triggers (20, 21, 22, 23, 24). In contrast, autosomal recessive OTULIN deficiency and dominant-negative OTULIN deficiency manifest with life-threatening autoinflammation (12, 13, 14, 17, 18). The late-onset phenotype in OTULIN haploinsufficiency may be overshadowed by the early-onset autoinflammatory manifestations in autosomal recessive and dominant-negative OTULIN deficiency (20). Clinical, immunological, biochemical, and molecular findings in patients suffering from the different forms of OTULIN deficiency suggest a disease spectrum, with OTULIN haploinsufficiency requiring exogenous triggers to induce hyperinflammation. Possibly, the nature and origin of these triggers contribute to the apparent phenotypic heterogeneity seen in OTULIN haploinsufficiency. One established trigger of necrosis in patients suffering from OTULIN haploinsufficiency is the staphylococcal α-toxin, acting through the cell type–dependent accumulation of caveolin-1 (20, 23). In the patients described here, *P*. *aeruginosa* was cultured from clinical specimens in three patients, and *C*. *perfringens* was isolated from clinical cultures in two patients. *C. perfringens* secretes multiple cytotoxins, some of which rely on caveolin-1 for their cytotoxic activity (40, 41). Apparently noninfectious triggers were described in a few cases reported earlier (20). The traumas reported in the current study, including vaccination procedures and elective surgeries (20, 21), indicate that also mechanical triggers are sufficient to induce necrotic and hyperinflammatory sequelae in OTULIN haploinsufficiency. Caveolin-1 controls wound healing by regulating cell proliferation and migration in the skin (42). Wound healing depends on a controlled inflammatory response (43, 44, 45), and the contribution of a defective wound healing to the induction of hyperinflammation in OTULIN haploinsufficiency remains to be established. Collectively, these observations point to the work of other, specific, microbial and nonmicrobial, triggers in addition to the staphylococcal α-toxin.

OTULIN haploinsufficiency is biochemically silent for TNF-induced signaling events, with OTULIN haploinsufficient cells displaying a normal cytokine response and a normal sensitivity to TNF-induced cell death. Nonetheless, the patients’ clinical response to immunomodulatory therapies suggests a role for these regimens in the treatment of their triggered necrosis and subsequent hyperinflammation. Although with no specific molecular target and with variable individual effects, patients with OTULIN haploinsufficiency respond to high doses of steroids during their episodes of necrotic and inflammatory disease. Experience with interleukin-1 receptor antagonists and Janus kinase inhibitors is limited and with unpredictable, but occasionally positive, results (46). To date, most consistent results using biological therapeutics have been obtained with TNF inhibitors, but the precise cellular source and pathophysiological role of TNF in OTULIN haploinsufficiency remain to be established. Immunological characterizations of OTULIN-haploinsufficient PBMCs have demonstrated the absence of overt immunological disturbances (20). The neutrophilic infiltration seen in the histopathological samples from some patients suggests a pathophysiological role for neutrophils. Damage-associated molecular patterns, released following the triggered necrosis in the skin and/or lungs and contributing to the recruitment of neutrophils, may provoke the subsequent release of TNF and the following inflammation that is seen in patients with OTULIN haploinsufficiency. Irrespective of the triggers of the disease, and with the endpoint being a hyperinflammatory state, the cases reported here indicate that OTULIN haploinsufficient patients benefit from immunomodulatory therapies in conjunction with antibiotics (21, 22).

## Materials and methods

### Human subjects

All investigations were conducted according to the principles expressed in the Helsinki Declaration. Informed consents were obtained in accordance with local regulations, and a protocol for research on human subjects was approved by the institutional review boards of Radboud University Medical Center (protocol NL40331.078), Institut National de la Santé et de la Recherche Médicale (protocol C10-16), The Rockefeller University (protocols JCA-0698 and JCA-0695), the University of British Columbia (protocols H18-02853 and H19-01657), and the National Institutes of Health (protocol 94-HG-105). Experiments were conducted in the United States, Canada, and the Netherlands, in accordance with local regulations and with the approval of The Rockefeller University, University of British Columbia, and University Medical Center Utrecht, respectively.

### Case reports

The clinical course of patient A.II.4 has been recently reported elsewhere (21). The patient, a female born to unrelated Dutch parents, was treated with immunoglobulins for a transient IgG2 deficiency during childhood. At the age of 4 years, the patient was admitted for antibiotic treatment of pneumonia and lung abscesses, but no pathogens were identified. At the age of 31 years and following a minor trauma of the skin, the patient was admitted to the ICU because for necrotizing cellulitis in conjunction with the need for hemodynamic and respiratory support. Surgery was performed, and pathological examination revealed neutrophilic infiltrates. No pathogenic microorganisms were cultured, and the patient responded insufficiently to surgery and antibiotics. During admission, the patient developed pathergy-like inflammation following minor traumas of the skin. Treatment with steroids and anakinra did not improve the clinical course, but the patient responded favorably after switching anakinra to infliximab, and the patient was discharged. Diagnostic WES was performed because of a suspicion for an IEI. Panel analysis of genes known to underlie IEIs revealed a heterozygous mutation in *OTULIN*, and no variants fitting the known modes of inheritance for any of the other genes analyzed. The patient’s OTULIN variant (p.A240V) was carried by her mother, who was asymptomatic. Diagnostic immunophenotyping in patient A.II.4 revealed no abnormalities and normal levels of immunoglobulins.

Patient B.III.8 is a 10-year-old boy from Iran who was born to non-consanguineous parents. At the age of 18 mo and shortly after receiving his measles, mumps, and rubella vaccination, the patient was admitted to the hospital because of necrotizing cellulitis with abscesses at the site of injection, requiring surgery. No cultures were taken before start of antibiotic treatment, and perioperative cultures returned growth of *P. aeruginosa*. Pathological examinations were suggestive of panniculitis. The necrotizing skin disease was ultimately controlled, and the patient was discharged. At the age of 6 years and 1 wk after receiving his diphtheria and tetanus vaccination, the patient was again admitted because of necrotizing cellulitis and abscesses at the site of injection. At other sites of the skin, the patient also manifested necrotizing panniculitis. The patient was treated with antibiotics, and surgical exploration was performed, ultimately resulting in resolution of the disease. Perioperative cultures returned *Enterobacter* species. As part of the national vaccination program, the patient had received other vaccinations without complications. No immunomodulatory therapy was given. WES revealed a heterozygous mutation in *OTULIN*. The patient did not carry variants fitting the known modes of inheritance for any of the other genes implicated in IEIs. Familial segregation identified three carriers of the OTULIN variant (p.E314X) across three generations (patients B.I.1, B.II.4, and B.III.7), who were all asymptomatic. Diagnostic immunophenotyping in patient B.III.8 revealed no abnormalities and normal levels of immunoglobulins. The activity of the complement system was normal, and dihydrorhodamine and nitroblue tetrazolium tests showed a normal oxidative burst in neutrophilic granulocytes.

Patient C.II.3 is a 16-year-old Argentinian male who was born to non-consanguineous parents. Between the ages of 6 and 18 mo and shortly after receiving vaccinations, the patient developed abscesses at the site of injection, requiring intravenous treatment with antibiotics. At the age of 18 mo, the patient was admitted for pneumonia and treated with antibiotics. A computerized tomography scan showed intraparenchymal bullae. The patient developed normally until the age of 16 years, when he underwent varicocelectomy. This procedure was complicated with the development of a necrotizing fasciitis with Fournier’s gangrene and septic shock, requiring emergency surgery and ICU admission. A catheter-related thrombophlebitis of the upper limb developed during admission, resulting in another episode of necrotizing fasciitis requiring surgery. In addition, the patient had multiple surgeries for the treatment of cavitating pressure ulcers. Cultures from the wounds returned *P. aeruginosa*, *S. aureus*, and *C. perfringens*, along with other bacterial and fungal flora for which the patient was treated with antimicrobials. Pathological examinations of the lesions were compatible with acute inflammation and showed neutrophilic infiltrates. During admission, the patient also developed a hospital-acquired pneumonia caused by *P. aeruginosa*. With the suspicion of a hyperinflammation state, immunomodulatory therapy was started, consisting of immunoglobulins and steroids in conjunction with adalimumab. The clinical response to this regimen allowed for discharge, although elevated inflammatory parameters (among of which the C-reactive protein and ferritin) persisted. Currently, the patient is treated with baricitinib, and his disease is controlled both clinically and immunologically. A WES-based panel analysis in the patient revealed a heterozygous mutation in *OTULIN*, but no variants fitting the known modes of inheritance for other genes implicated in IEIs. The patient’s OTULIN variant (p.K247RfsX25) was carried by his father and sister, who were both asymptomatic. Diagnostic immunophenotyping in patient C.II.3 revealed no abnormalities and normal levels of immunoglobulins. The activity of the complement system was normal, and a dihydrorhodamine test showed a normal oxidative burst capacity.

Patient D.II.3, a 28-year-old previously healthy female who was born to non-consanguineous German parents, developed surgical site abscesses with necrotizing cellulitis following elective reduction mammoplasty. No pathogens were identified, despite culturing of multiple samples. Pathological examinations of the skin showed granulating tissue but with no mononuclear and histiocytic infiltrates or other specific characteristics. Because the skin lesions were insufficiently responsive to revision surgery and treatment with antibiotics, a hyperinflammatory disease was suspected, and steroids in conjunction with anakinra were initiated. This immunomodulatory therapy resulted in a clinical improvement with resolution of the disease, and the steroids and anakinra were tapered over time. Diagnostic WES-based panel analysis of known to underlie IEIs revealed a heterozygous mutation in *OTULIN*. Segregation analyses could not be performed to confirm inheritance of the patient’s OTULIN variant (p.S351VfsX55). Diagnostic immunophenotyping in the patient revealed no abnormalities. WES-based panel analysis in the patient also identified a heterozygous missense variant of unknown significance (p.C20R, with a MAF of 1 × 10^−04^, a CADD score of 13, and a REVEL score of 0.153) in *TNFRSF18B*, but routine diagnostics revealed normal levels of immunoglobulins.

The clinical course of patient E.II.8 has been recently reported elsewhere (22). The patient, born to Spanish parents, presented from the age of 15 years onward with episodes of cellulitis requiring surgery following minor traumas of the skin. Cultures taken from the wound returned *Pseudomonas* species and *Klebsiella* species once but remained negative at other occasions. At the age of 27 years and while pregnant, the patient suffered from a septic shock following a chorioamnionitis caused by *C. perfringens*. In the years following, the patient was admitted and treated with antibiotics for recurrent episodes of pyelonephritis caused by *Escherichia coli*. Structural abnormalities of the urinary tract were excluded. No immunomodulatory therapy was given. Diagnostic analysis with a next-generation sequencing–based IEI-targeted panel demonstrated a heterozygous mutation in *OTULIN*. Segregation analyses showed four carriers of the patient’s OTULIN variant (p.W148X) across three generations (patients E.I.2, E.II.4, E.II.7, and E.III.10). Patients E.I.2 and E.II.4 were asymptomatic, but patients E.II.7 and E.III.10 appear mildly symptomatic by presenting episodes of cellulitis. Diagnostic immunophenotyping in patient E.II.8 revealed no abnormalities and normal levels of immunoglobulins. The activity of the complement system was normal, and no markers of autoimmunity were detected.

Patient F.II.3, a now 43-year-old male who was adopted and whose family history is unknown, presented at 2 mo of age with a right axillary abscess at the site of BCG vaccine requiring drainage. This was followed by recurrent soft tissue abscesses, often occurring at the site of prior injury, surgery, immunization, or insect bite, and responsive to corticosteroids. This included one episode close to 3 years of age, where after sustaining trauma to his head, he developed a fluctuant mass that progressed to necrosis of most of the scalp layers, including periosteum, with histology demonstrating marked infiltration of neutrophils. Culture grew *Staphylococcus epidermidis*, deemed a contaminant of the open head wound, while cultures from other abscesses were negative. He had one episode of pneumonia and osteomyelitis in childhood and was admitted in adolescence for presumed gastrointestinal infection that responded to corticosteroids. The patient was ultimately diagnosed with inflammatory bowel disease at age 27 after presenting with fever and abdominal pain. Due to concern for appendicitis, he underwent an appendectomy and small bowel resection, with pathology demonstrating chronic active ileitis and perforation. The episodes of recurrent skin and soft tissue inflammation have decreased in frequency into adulthood, occurring every 3–5 years. WES demonstrated a heterozygous *OTULIN* variant (NM_138348.6: c.788G>A,). Diagnostic immunophenotyping was normal, and the patient had normal immunoglobulin levels apart from a mildly low IgM level.

### WES

WES was performed on the patients’ genomic DNA, obtained from peripheral blood, in the referring centers as part of routine diagnostics (27). Genomic *OTULIN* variants in patients and relatives were validated by amplifying 200- to 300-bp regions encompassing the mutation from gDNA samples with site specific primers. The amplicons were then sequenced with BigDye Terminator technology on an ABI 3730 DNA sequencer. SnapGene (version 8.0.0) was used for sequence analysis.

### Cell culture

PDFs were obtained from skin biopsy specimens and cultured in Dulbecco’s modified Eagle’s medium (DMEM), supplemented with GlutaMAX (Gibco), 10% heat-inactivated fetal bovine serum (HI-FBS) (Gibco), and 25 mM HEPES (Gibco) unless otherwise specified. PBMCs were isolated from whole blood by density gradient centrifugation on Ficoll-Paque PREMIUM (Cytiva) and maintained in Roswell Park Memorial Institute 1640 medium (Gibco), supplemented with 10% HI-FBS or cryopreserved. T cells were isolated from cryopreserved PBMCs by using the Essay Step Human T cell isolation kit (Stem Cell Technologies). Isolated T cells were cultured in IMC10 medium, consisting of IMC supplemented with 10–50 U/ml IL-2 and CD3/CD28 activator (25 μl/ml final concentration). HEK293T cells (American Type Culture Collection, RRID:CVCL_0063, not authenticated) were cultured in DMEM, supplemented with GlutaMAX, 10% HI-FBS, and 25 mM HEPES. All cell lines were tested for mycoplasma contamination using the MycoAlert Mycoplasma Detection Kit (Lonza Bioscience) and used in experiments exclusively in case no contamination was detected.

### Generation of OTULIN constructs and transfection

The human canonical *OTULIN* cDNA open reading frame clone was amplified from the pCMV6-AN-Myc-DDK-FAM105B (20). To generate the *OTULIN* W148X, A240V, K247RfsX25, and E314X variants, we performed a modified overlap-extension PCR-based method (Table S4) (20, 47). The *OTULIN* S351VfsX55 variant was generated by restriction digestion of the human canonical *OTULIN* cDNA open reading frame with XagI and NotI, and the subsequent ligation with a gBlock (Table S4) containing the out-of-frame sequence and new stop codon (Integrated DNA technologies) through Gibson assembly (NEB). All constructs were validated by Sanger sequencing. HEK293T cells were transiently transfected in the presence of Lipofectamine LTX (Thermo Fisher Scientific).

### Production of recombinant α-toxin

The α-toxin gene of *S. aureus* strain Newman was synthesized and modified by addition of a C-terminal cysteine, followed by a thrombin cleavage site and a His-tag. This was cloned into the pET28A vector (Novagen) and transformed into *E. coli* BL21(DE3) (Thermo Fisher Scientific) (Table S4). For a typical sample preparation, bacteria were grown in Luria Broth at 37°C until A_600_ of 0.5, prior to addition of 1 mM isopropyl β-d-thiogalactopyranoside to induce protein expression. After 4 h, cells were harvested by spinning at 4°C for 15 min at 3,500 × *g*. The supernatants were discarded, and pellets were directly lysed through resuspension in a denaturing lysis buffer (50 mM Tris, 500 mM NaCl, and 6 M guanidine hydrochloride, pH 8) and sonification. His-tagged α-toxin was isolated using 5 ml HisTrap HP (Cytiva) columns according to the manufacturer’s protocol and subsequently dialyzed into PBS.

### NF-κB inhibition assay

HEK293T cells were seeded in 96-well plates coated with 0.01% poly-L-lysin (Sigma-Aldrich). After 24 h, the cells were co-transfected with pCMV6 vectors containing the *OTULIN* variants, the NF-κB response element-driven *luc2P* luciferase vector pGL4.32 (Promega), and the *Renilla* luciferase vector (pRL-TK) (Promega) as an internal control. 24 h after transfection, the cells were stimulated with 20 ng/ml recombinant human TNF-α (R&D Systems) in DMEM, supplemented with GlutaMAX, 1% HI-FBS, and 25 mM HEPES, for an additional 24 h. The ratio of firefly-to-*Renilla* luciferase expression was assessed with the Dual-Glo Luciferase assay (Promega) on the CLARIOstar Plus plate reader (BMG Labtech). The data were normalized against the inhibitory activity of cells transfected with an empty pCMV6 vector (as background) and of cells transfected with the pCMV6 vector containing the *OTULIN* reference cDNA.

### Whole-cell lysates, SDS-PAGE, and western blotting

HEK293T cells in a 6-well plate were transiently transfected with the pCMV6 vectors containing the *OTULIN* variants for 24 h. PDFs in a 6-well plate were synchronized by incubation in DMEM supplemented with GlutaMAX, 1% HI-FBS, and 25 mM HEPES for 24 h. T cells were expanded in ImmunoCult-XF T Cell Expansion Medium (Stem Cell Technologies) and incubated overnight before lysing. Samples of HEK293T and PDFs, as well as expanded T cells and freshly thawed PBMCs, were split in two and subsequently lysed in either RLT lysis buffer (Qiagen) supplemented with 0.14% β-mercaptoethanol (for later RNA isolation) or radioimmunoprecipitation assay buffer (50 mM Tris, NaCl 150 mM, 1% NP40, and 0.1% sodium dodecyl sulfate) supplemented with the cOmplete mini and EDTA-free Protease Inhibitor cocktail (Roche) (for western blotting). All lysates were cleared through sonication and centrifugation and subsequently stored at −80°C until further use. Laemmli buffer (Bio-Rad) supplemented with 100 mM dithiothreitol (DTT) was added to the clarified lysates, which was then boiled. Proteins were separated on 4–20% Mini-PROTEAN TGX gels (Bio-Rad) and transferred onto a 0.2-µm polyvinylidene difluoride (PVDF) membrane (Bio-Rad). The membrane was blocked with PBS supplemented with 0.1% Tween and 5% bovine serum albumin, incubated overnight with the primary antibody, followed by the appropriate horseradish peroxidase–conjugated secondary antibody. The primary antibodies used were as follows: anti-OTULIN rabbit polyclonal antibody (1:1,000, Cat# ab151117; Abcam, RRID:AB_2728115), anti-HOIP rabbit polyclonal antibody (1:1,000, Cat# ab46322; Abcam, RRID:AB_945269), anti–M1-Ub mouse monoclonal antibody (1:5,000, Cat# MABS451; Millipore, RRID:AB_2929000), anti–Caveolin-1 rabbit monoclonal antibody (1:1,000, Cat# 3267; Cell Signaling Technology, RRID:AB_2275453), and anti–β-actin mouse monoclonal antibody (1:1,000, Cat# AM1829b; abcepta, RRID:AB_10664137). The secondary antibodies used were HRP-conjugated anti-rabbit IgG goat polyclonal antibody (1:10,000, Cat# 4030-05; SouthernBiotech, RRID:AB_2687483) and HRP-conjugated anti-mouse IgG1 goat polyclonal antibody (1:10,000, Cat# 1070-05; SouthernBiotech, RRID:AB_2650509). The proteins were visualized by enhanced chemiluminescent staining using ECL reagents (Thermo Fisher Scientific). Band intensity analysis was performed with ImageJ or Empiria Studio (LI-COR Biosciences).

### Reverse transcription and real-time PCR

Total RNA was extracted from the transiently transfected HEK293T cells and PDFs using the RNeasy Mini Kit (Qiagen), whereas the total RNA from the PBMCs was isolated with the RNeasy Micro Kit (Qiagen). Reverse transcription was performed with the SuperScript III first-Strand Synthesis System (Invitrogen), using random hexamers as the primers. The TaqMan Universal PCR Master Mix (Applied Biosystems) and the TaqMan Gene Expression assays (Thermo Fisher Scientific) for *OTULIN* (Hs01113237_m1) and *GUSB* (Hs00939627-m1) were used for the quantitative PCR, which was performed in the StepOnePlus Real-Time PCR System (Applied Biosystems) using the cycling parameters recommended by the manufacturer. Relative expression analyses were performed by the ΔCt method, using the StepOne Software v2.3 (Applied Biosystems). mRNA was isolated from blood using Tempus Spin RNA Isolation Kit (Invitrogen) and converted to cDNA using an iScript cDNA synthesis kit (Bio-Rad Laboratories). Primers used were: forward primer 5′-CAT​ACG​GCG​AGT​CCG​TGG​TG-3′ (exon 4) and reverse primer: 5′-CCT​CAC​ACA​CTC​TGA​CGG​GGA​TG-3′ (exon 7). After loading on agarose gel, DNA was extracted from excised bands and followed by Sanger sequencing.

### Cell viability assays

To determine patient PDFs’ sensitivity to TNF, PDFs in a 96-well plate were synchronized in DMEM supplemented with GlutaMAX, 1% FBS, and 25 mM HEPES for 24 h. Cells were subsequently stimulated with 20 ng/ml recombinant human TNF-alpha and 12.5 ng/ml cycloheximide at 37°C and 5% CO_2_. For the α-toxin cytotoxicity assay, PDFs in a 96-well plate were synchronized for 24 h in DMEM supplemented with GlutaMAX, 1% FBS, and 25 mM HEPES, which had been set to a pH of 6.0 through titration with HCl, at 37°C and 5% CO_2_. After, cells were intoxicated with α-toxin in dose response with a maximum concentration of 30 µg/ml. After 24 h of stimulation or intoxication, cell viability was assessed with the CellTiter-Glo Luminescent Cell viability Assay (Promega). Luminescence was measured on the CLARIOstar Plus plate reader. The half-maximum effective concentrations were determined through three-parameter nonlinear regression analyses.

### Cytokine expression assays

PDFs were seeded at a density of 2 × 10^5^ cells per well in a 48-well flat-bottomed plate in DMEM containing 10% FBS. 4 h after seeding, the medium was replaced with DMEM containing 1% FBS. 24 h after seeding, the cells were stimulated with 20 ng/ml of TNF (210-TA-005; R&D Biosystems). 24 h after stimulation, the supernatants were recovered and stored at −80°C. The levels of IL-6 and IL-8 were assessed with the IL-6 and IL-8 Human enzyme linked immunosorbent assay (ELISA) kits (88706688 and 88808688; Invitrogen, respectively) in accordance with the manufacturer’s protocol.

### In silico analyses of predictors of OTULIN variant deleteriousness

For the development of an in silico pipeline to predict the deleteriousness of OTULIN variants, we used the experimentally derived allele activities of coding sequence variants reported elsewhere (20) and those acquired in the present study. Simple linear regression analyses were performed using GraphPad Prism, version 10.5.0. For the regression analysis with the MAF scores, the allele activities of all experimentally tested OTULIN variants were used. For the regression analysis with CADD, AlphaMissense, SIFT, PolyPhen-2, REVEL, and MutationAssessor scores, only the allele activities of missense variants with a MAF <1 × 10^−5^ were included. Principal component analyses, optimal cut point calculations, and receiver operating characteristic (ROC) curve analyses were performed using RStudio version 2025.05.1, using the factoextra (48) and cutpointr (49) packages. For the MAF, REVEL, and AlphaMissense cut point calculations, OTULIN variants with allele activities below or equal to 25% were labeled as deleterious. All data used in in silico analyses are available in Table S3.

### Online supplemental material

The supplemental information related to this manuscript contains Figs. S1, S2, S3, and S4 and Tables S1, S2, S3, and S4. Fig. S1 shows the population genetics metrices of missense variants of *OTULIN* reported in the gnomAD database. Fig. S2 shows the absence of negative dominance of patients’ *OTULIN* alleles. Fig. S3 shows the sensitivity of patients’ PDFs to the staphylococcal α-toxin in relation to the abundance of caveolin-1. Fig. S4 shows ROC curves for the prediction of OTULIN variant deleteriousness. Table S1 contains the data on routine diagnostic immunological characterizations. Table S2 contains the data on the clinical responses to antimicrobial and immunomodulatory therapies. Table S3 contains the data of allele activities and in silico predicted deleteriousness of *OTULIN* alleles. Table S4 provides an overview of the primers and gBlocks used for this study.

## Supplementary Material

Table S1 shows the routine diagnostic immunological characterizations.

Table S2 shows the clinical responses.

Table S3 shows the allele activities and in silico predicted deleteriousness of *OTULIN* alleles.

Table S4 shows the primers and gBlocks used.

SourceData F2 is the source file for Fig. 2.

SourceData F3 is the source file for Fig. 3.

SourceData F4 is the source file for Fig. 4.

SourceData F5 is the source file for Fig. 5.

## Data Availability

All experimental data in this study are available in the article and its supplemental materials. The WES data underlying this study are not publicly available for reasons of patient privacy. The WES data are available from the corresponding author upon reasonable request and upon establishment of a data transfer agreement.
