# Supplementary figures and images for: Novel genotypes, phenotypes, and triggers in humans with OTULIN haploinsufficiency
Source: J Hum Immun. 2025 Sep 30;1(4):e20250018. doi: 10.70962/jhi.20250018 (PMC12643120; doi:10.70962/jhi.20250018)

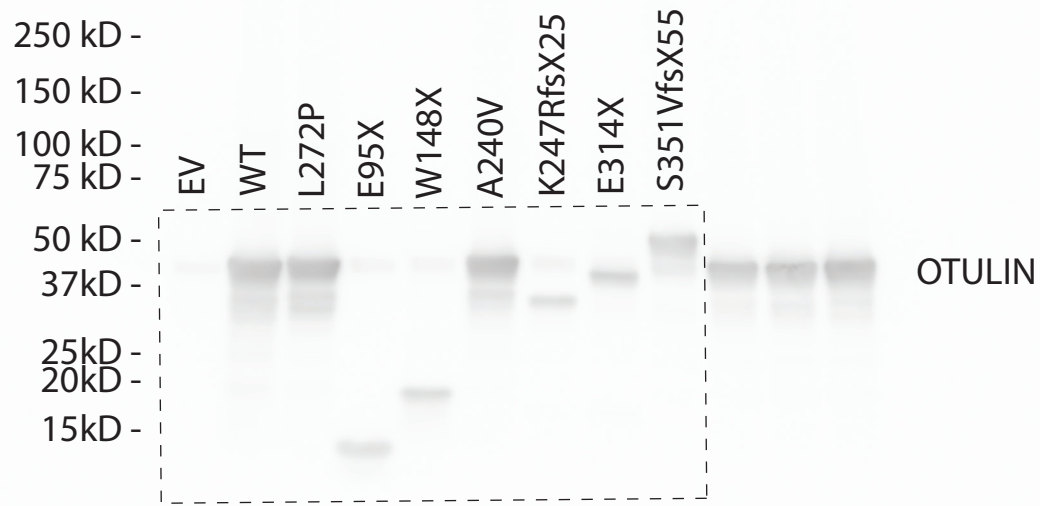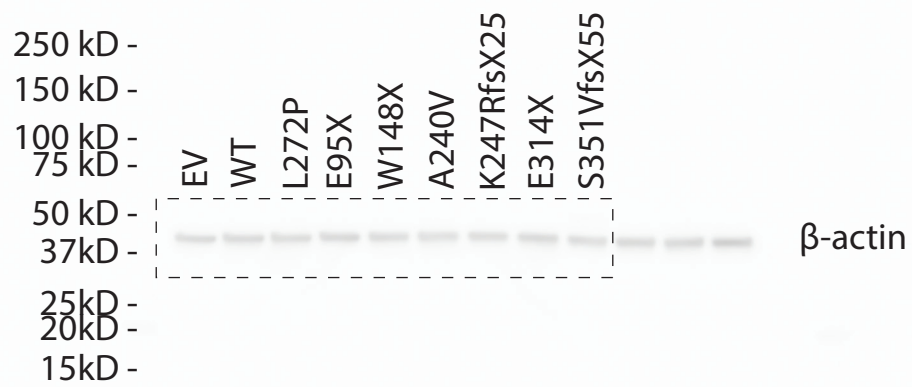

Supplement: SourceData F2 — is the source file for Fig. 2. [file jhi_20250018_sourcedataf2.pdf]

Figure 4A:

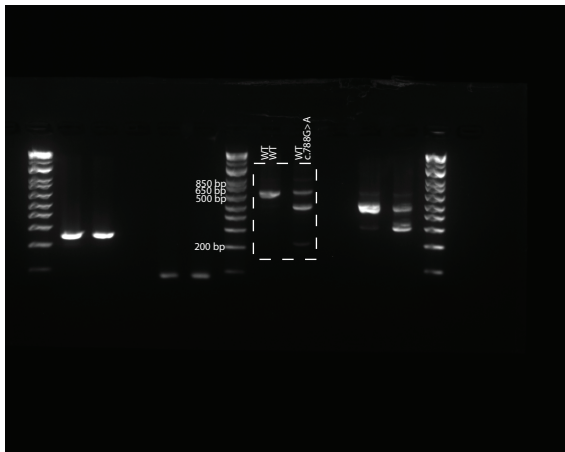

Figure 4C:

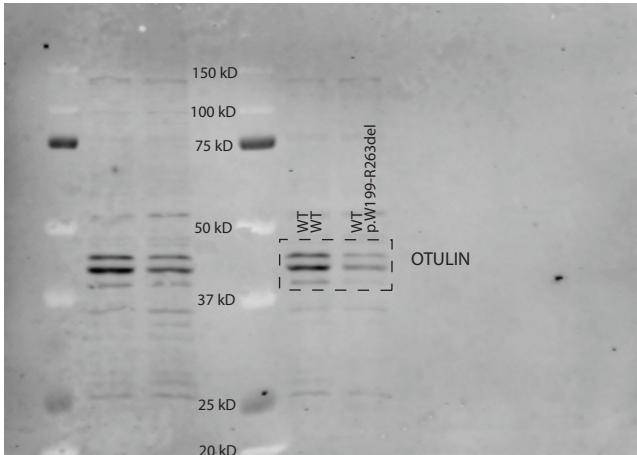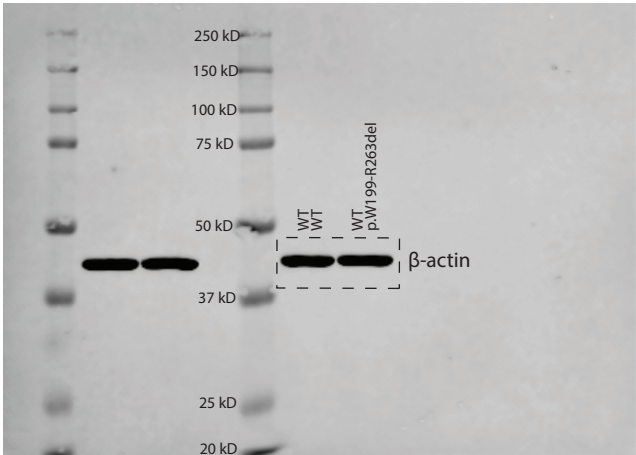

Supplement: SourceData F4 — is the source file for Fig. 4. [file jhi_20250018_sourcedataf4.pdf]

Figure 5A:

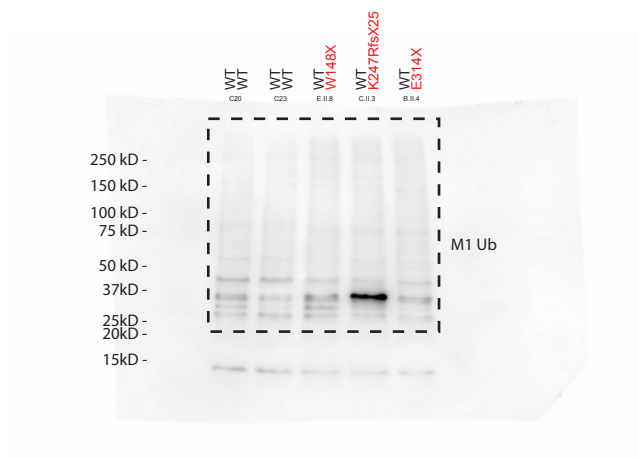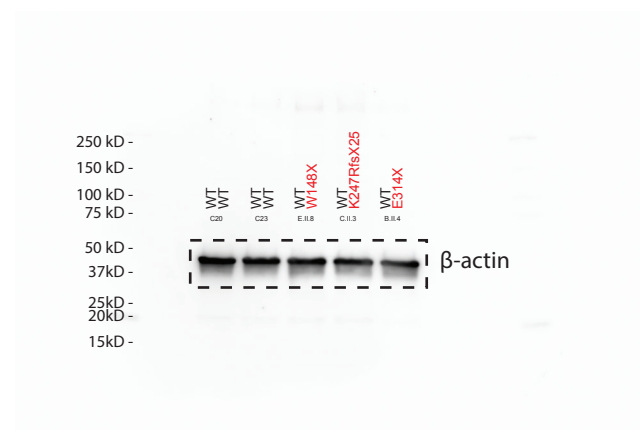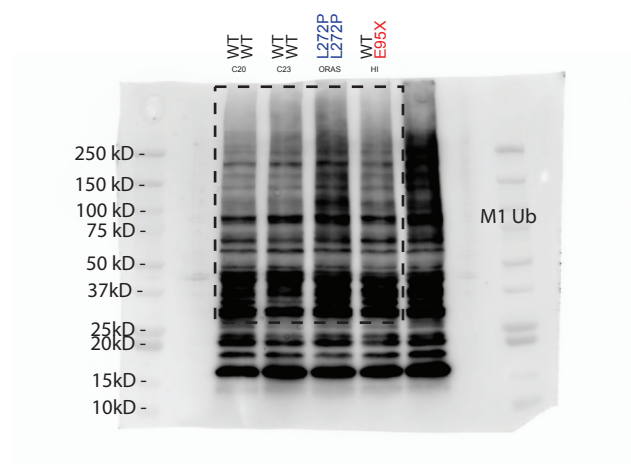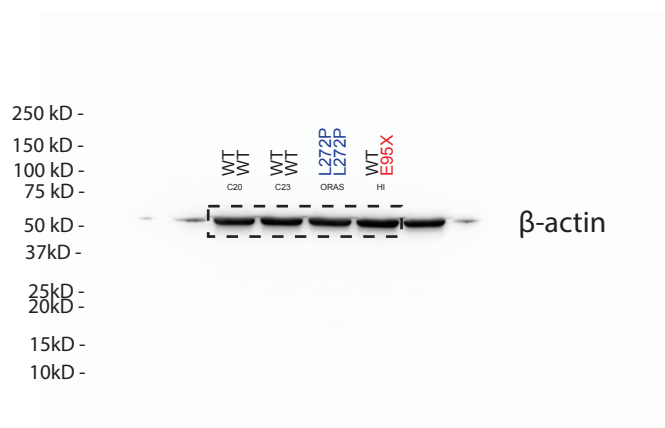

Figure 5E:

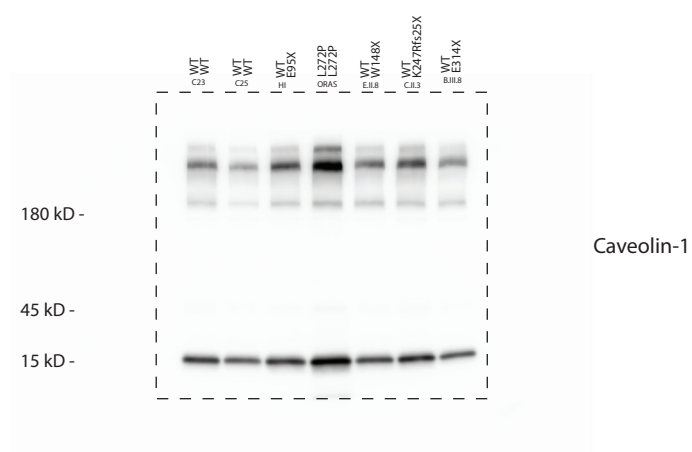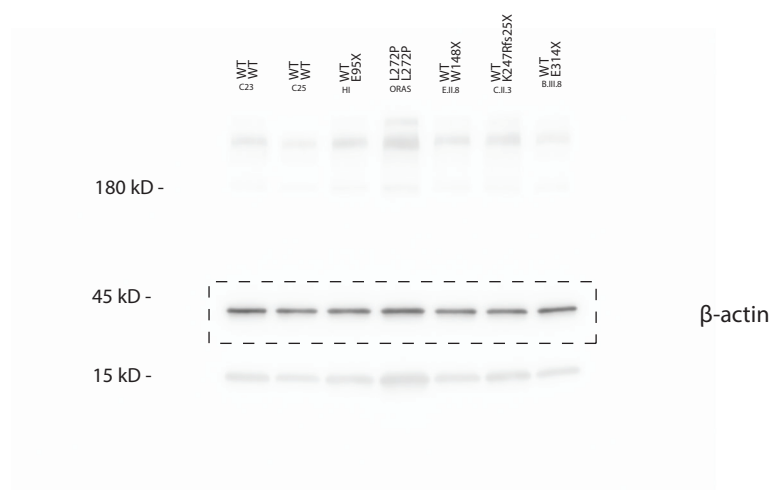

Supplement: SourceData F5 — is the source file for Fig. 5. [file jhi_20250018_sourcedataf5.pdf]
