# Supplementary material for: Novel genotypes, phenotypes, and triggers in humans with OTULIN haploinsufficiency
Source: J Hum Immun. 2025 Sep 30;1(4):e20250018. doi: 10.70962/jhi.20250018 (PMC12643120; doi:10.70962/jhi.20250018)
Supplement: SourceData F3 — is the source file for Fig. 3. [file jhi_20250018_sourcedataf3.pdf]

Figure 3B:

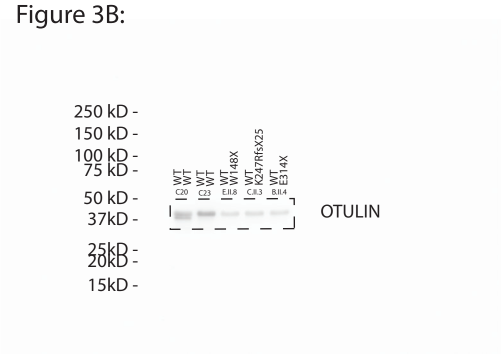

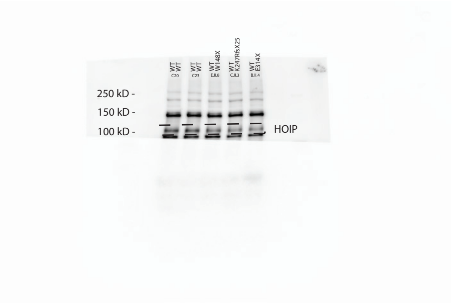

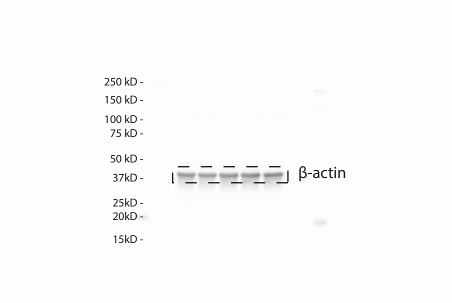

Figure 3D:

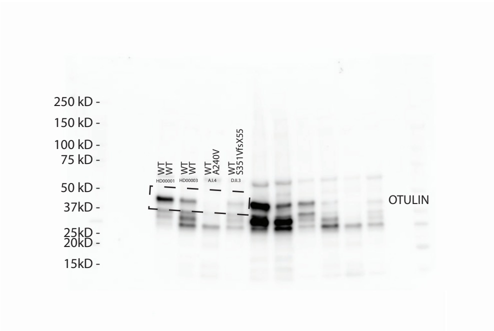

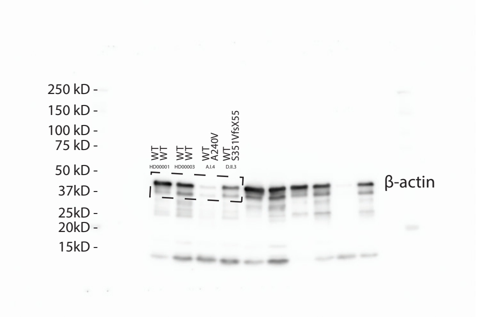

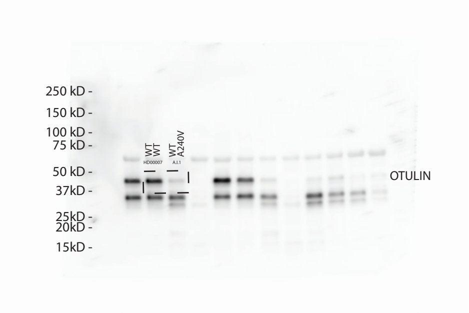

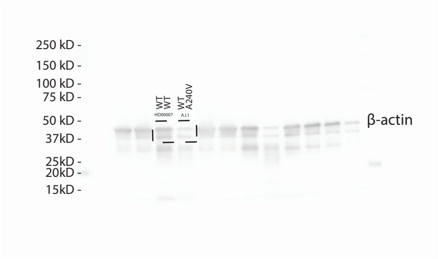

SourcedataF3
